# Sound source localization patterns and bilateral cochlear implants: Age at onset of deafness effects

**DOI:** 10.1371/journal.pone.0263516

**Published:** 2022-02-08

**Authors:** Sean R. Anderson, Rachael Jocewicz, Alan Kan, Jun Zhu, ShengLi Tzeng, Ruth Y. Litovsky

**Affiliations:** 1 Waisman Center, University of Wisconsin-Madison, Madison, Wisconsin, United States of America; 2 Department of Audiology, Stanford University, Stanford, California, United States of America; 3 School of Engineering, Macquarie University, New South Wales, Australia; 4 Department of Statistics, University of Wisconsin-Madison, Madison, Wisconsin, United States of America; 5 Department of Mathematics, National Sun Yat-sen University, Kaohsiung, Taiwan; University of Texas at Dallas, UNITED STATES

## Abstract

The ability to determine a sound’s location is critical in everyday life. However, sound source localization is severely compromised for patients with hearing loss who receive bilateral cochlear implants (BiCIs). Several patient factors relate to poorer performance in listeners with BiCIs, associated with auditory deprivation, experience, and age. Critically, characteristic errors are made by patients with BiCIs (e.g., medial responses at lateral target locations), and the relationship between patient factors and the *type* of errors made by patients has seldom been investigated across individuals. In the present study, several different types of analysis were used to understand localization errors and their relationship with patient-dependent factors (selected based on their robustness of prediction). Binaural hearing experience is required for developing accurate localization skills, auditory deprivation is associated with degradation of the auditory periphery, and aging leads to poorer temporal resolution. Therefore, it was hypothesized that earlier onsets of deafness would be associated with poorer localization acuity and longer periods without BiCI stimulation or older age would lead to greater amounts of variability in localization responses. A novel machine learning approach was introduced to characterize the types of errors made by listeners with BiCIs, making them simple to interpret and generalizable to everyday experience. Sound localization performance was measured in 48 listeners with BiCIs using pink noise trains presented in free-field. Our results suggest that older age at testing and earlier onset of deafness are associated with greater average error, particularly for sound sources near the center of the head, consistent with previous research. The machine learning analysis revealed that variability of localization responses tended to be greater for individuals with earlier compared to later onsets of deafness. These results suggest that early bilateral hearing is essential for best sound source localization outcomes in listeners with BiCIs.

## Introduction

Cochlear implants (CIs) are designed to provide access to auditory information and to improve speech understanding for individuals with severe-to-profound hearing loss. The addition of a second CI has the potential to provide benefits stemming from binaural hearing, such as localization of sounds sources in space, improved speech understanding in noise, and reduction in listening effort [1–3]. However, outcomes vary widely from patient-to-patient. The present paper focuses on sound source localization and the factors that affect localization performance in patients with bilateral CIs (BiCIs).

There is extensive literature documenting the impact of patient-dependent factors on localization error for listeners with BiCIs. Several factors associated with auditory deprivation and experience with BiCIs are predictive of sensitivity to cues used for localization, outlined by Thakkar and colleagues [4]. Age at onset of deafness [5–11], the duration of experience with unilateral or bilateral, acoustic or CI stimulation [5, 6, 8, 10, 12–14], and age at testing [15–17] are predictive of localization performance in children and adults. The goal of the present experiment was to determine the contribution of these factors to sound source localization by comparing differences in the pattern of localization responses.

The process of sound source localization must require at least two steps: (1) encoding of binaural cues used to localize, and (2) decoding of neural responses, assigning them to locations in space. Binaural cues include interaural time and level differences (ITDs and ILDs). These cues are conveyed in the temporal fine-structure (instantaneous changes in pressure or intensity over time) and temporal envelope (slower, more gradual changes in pressure or intensity over time) of a signal. Both encoding and decoding could contribute to the type of localization errors made by different listeners with BiCIs. Specifically, encoding is thought to occur in binaural nuclei in the brainstem where the processing could be compromised with anatomical reorganization (for review, see [6, 18]), or deterioration of inputs due to factors associated with prolonged deafness (for review, see [19]) and older age during adulthood (for review, see [20]). Poorer temporal fidelity due to peripheral degradation may be similar to the introduction of decorrelation between the sounds presented to both ears, resulting in more variable responses from binaural nuclei, and a wider sound image [21] or less reliability from trial to trial. Decoding is less understood. One thing is clear: BiCI experience plays a role as training can refine strategies used for localization [22–25]. Thus, problems with decoding may be more highly associated with systematic errors in a listener’s responses.

### Differences in localization performance between listeners

Assuming that bilateral auditory deprivation, experience, and age can affect the encoding and decoding of binaural cues, one should also expect that these factors will affect the ability to accurately localize sound sources. Most studies assess localization via the average error across presentation, or target, angles using root-mean-square (RMS) error. One challenge is that many studies of adults and children with BiCIs have exhibited vast amounts of variability across listeners, even with similar hearing histories (e.g., [3, 13, 26–31]).

**Fig 1:**
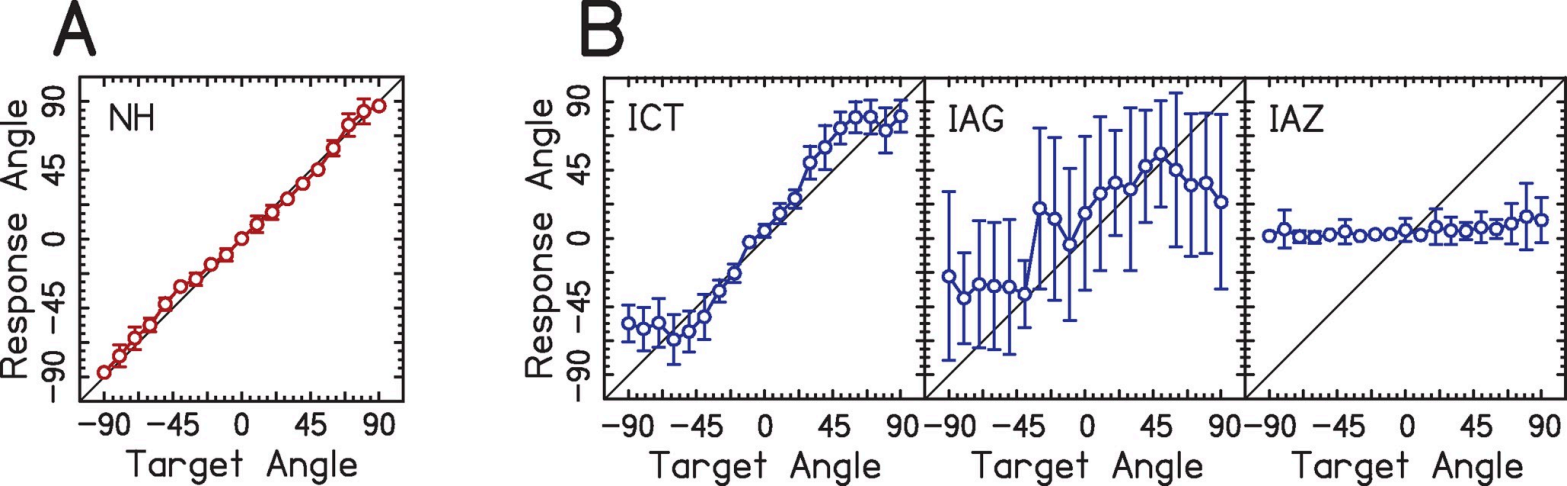
Example data from listeners with NH and BiCIs. The x-axis shows the angle from which the target speaker presented pink noise bursts. The y-axis shows the angle from which the listener perceived the sound. The diagonal shows perfect performance. Open circles represent mean response angles and error bars represent ±1 standard deviation. (A) Data from one listener with NH. (B) Data from three listeners with BiCIs whose subject codes are given in the top-left portion of each panel. Listener IAG’s errors were driven by variability in their responses across all target speakers. Listener IAZ’s errors were driven by highly consistent biases toward the front/center speaker for lateral target speakers.

Some examples of the differences in localization performance across listeners are shown in Fig 1 for a pink noise stimulus. Typical results from one example listener with normal hearing (NH) are shown on the left, and those from three different listeners with BiCIs are shown on the right. Fig 1 shows that the listener with NH has little or no bias in their responses (mean responses follow the main diagonal), and gives reliable responses (small standard deviation around mean), resulting in a small amount of RMS error. This pattern of localization responses is commonly observed in listeners with NH using wideband stimuli [32]. However, three very different localization functions are shown for the listeners with BiCIs. Listener with subject code ICT performs somewhat like the listener with normal hearing, but with larger errors at the lateral angles. Listeners IAG and IAZ have very similar amounts of RMS error (55.59 and 53.09 degrees, respectively), but the pattern of errors made from both listeners is very different. Responses from listener IAG are highly variable from trial to trial but average out to the accurate location, exhibiting little bias. However, listener IAZ has the opposite problem. They reliably report the sound source in the center speaker location, exhibiting a large amount of bias, but high reliability. Critically, the difference between listener IAG and IAZ would be missed if one only considers RMS error. This difference in patterns of localization responses between listeners IAG and IAZ is only obvious when the pattern of localization responses (response vs. target angle) is considered. Differences in response patterns may reflect the strategies used by listeners to overcome challenges due to deafness when spatial mapping abilities are being established.

To our knowledge, very few previous reports have addressed differences in the pattern of localization responses for listeners with BiCIs. Zheng and colleagues [22] and Grieco-Calub and Litovsky [10] described different patterns of localization responses in children with BiCIs. The authors suggest a hierarchy of performance and change in localization “categories.” That is, some patients cannot localize sounds, some are only able to discriminate between sound in the left vs. right frontal hemifields, and some are able to identify locations within a hemifield. Zheng and colleagues [22] developed the localization sensitivity index (LSI) as a statistical measure of sensitivity by assessing the pattern of localization responses, though this statistic has not been used outside of the initial report. In adults with BiCIs, Jones and colleagues [27] evaluated the amount of error at each angle of presentation using a cubic function across absolute target angle. Majdak and colleagues [31] evaluated the bias toward a particular side as well as the degree of errors that occurred within ±20 degrees to capture differences across individuals. Grantham and colleagues [3] reported the range of mean localization responses. Seeber and Fastl [33] and Schoen and colleagues [30] fit localization responses with a regression based on target locations and evaluated the slope and deviation of the regression line. Nopp and colleagues [13] assessed the deviance from ideal performance (the diagonal shown in Fig 1). Thus, no consensus has been reached across studies as to which metric is most appropriate and broadly applicable. In addition, it is important to better understand the relevance of statistical tools used to distinguish between patterns of localization responses to patients’ localization performance in the real world. In listeners with NH, the history of different measures of localization performance associated with different patterns is extensive. The major criteria used to assess NH localization performance are reviewed by Yost and colleagues [32], and suggest that for stimuli with a wide bandwidth, RMS error is appropriate for capturing performance. Some factors that result in significant changes in patterns of localization patterns of listeners with NH and hearing loss who use hearing aids are: (1) sound frequency [32, 34], especially for narrowband stimuli like sinusoids, (2) listening with one or both ears [35], (3) range of speakers used (i.e., “edge effects”) [36], (4) perturbation of ITD or ILD [37], and (5) use of different hearing devices [38].

One of the challenges to exploring the relationship between patient-dependent factors and pattern of localization responses is that either few data points (e.g., five repetitions per speaker, with 10 or fewer target angles) are collected from many (e.g., more than 50) participants [12, 39], or vice versa (e.g., 15 repetitions per speaker, with more than 10 target angles, and less than 10 participants) [3, 13, 14, 26–28, 30, 40–42]. To determine the pattern of localization responses, sufficient sampling of the localization function is necessary from each listener. Further, in order to statistically detect effects of patient-dependent factors, data from large number of listeners is needed due to large variability that is known to occur across listeners with CIs. Thus, statistical tools that are used to determine, classify, and otherwise characterize the patterns of localization responses should take into account differences in sample size, both within- and across-listeners.

It is highly likely that localization performance is limited by many different challenges, so large sample size studies are especially useful. For example, patient-dependent factors should be considered in light of the information provided to the auditory system by BiCI processors. CI processing removes fine-structure ITDs from the signal [43]. The use of high-rate electrical pulse trains with CI processing would limit the utility of ITDs in the fine-structure even if they were available [44, 45]. Thus, only ITDs in the envelope and ILDs are delivered to the patient. Further, clocks for both CI processors are independent and can introduce spurious or consistent and inaccurate interaural timing differences [46]. For these reasons, listeners with BiCIs weight ILDs much more heavily than ITDs when localizing sound sources [33, 41]. However, independently operating compression algorithms such as automatic gain control can result in the introduction of spurious interaural level differences [47, 48]. Microphone position has been associated with the characteristic errors like the ones observed by listener ICT [49, 50], suggesting that microphone position could be a major contributor to this characteristic localization response. Thus, even if a normally developing auditory system were stimulated with BiCIs, localization would be less accurate than with acoustic hearing.

### Study aims and hypotheses

Studies concerning binaural processing in children and young animals have shown that both encoding (activity-dependent anatomical reorganization in the brainstem; [18, 51]) and decoding (plasticity corresponding to age-dependent changes in head size; [52]) could be affected by the age at onset of deafness. In contrast, longer durations of deafness in one ear that occur in adulthood would primarily affect encoding by deteriorating peripheral portions of the binaural pathway.

The goal of the present study was to relate changes in degree of localization error and pattern of the localization responses with patient-dependent factors in adults with BiCIs. To address this goal, we used four different indices of sound source localization performance intended to capture the type and degree of error as well as the resulting pattern of localization responses. We evaluated several patient-dependent factors related to auditory deprivation and experience with BiCIs, as well as the age of participants.

Two related hypotheses were formulated. It was first hypothesized that if listeners had an earlier onset of deafness, then they would exhibit poorer localization performance because they have a sub-optimal map of binaural cues onto physical space (e.g., response means that show: high accuracy for central target locations and inability to distinguish lateral locations, only distinguish left from right, inability to judge left from right). It was further hypothesized that if listeners had a longer period of auditory deprivation, less BiCI experience, or were older at testing, then they would exhibit greater variability in their responses due to the introduction of decorrelation of neural signals from either ear sent to binaural nuclei in the brainstem.

## Materials and methods

### Testing procedures

#### Listeners

A total of 48 listeners with BiCIs participated in the present study (age 18–85 years; mean age 55 years). Their demographic information is listed in Table 1. Of those listeners, 44 were implanted during adulthood. All listeners had at least 11 months of listening experience with their CIs. Data from eight younger adult listeners with NH who were presented with the same stimuli were included as a point of comparison. All testing procedures were approved by the Health Sciences Institutional Review Board at the University of Wisconsin-Madison (submission number 2015-1438-CR005). Before participation began, listeners provided written informed consent.

**Table 1:** Demographic information for listeners with BiCIs.

Subject Code	Age at Onset (yrs)	Age at Testing (yrs)
IAG	0	56.25
IBA	0	74.92
IBN	0	64.92
ICW	0	21.33
IDD	0.5	18.17
IDF	1	18.75
ICG	2	50.08
ICH	2	32.42
ICZ	2.5	20.83
IAU	3	66.75
ICD	3	54.42
ICL	3	45.42
ICQ	3.83	19.25
ICO	4	32.83
ICP	4	50.67
IAJ	5	66.17
IBJ	8	30.67
ICX	8	74.42
IDA	8	48.67
ICC	9	66.75
ICN	10	40
ICA	13	52.25
IDB	17.5	71.33
ICT	18.5	21.33
ICB	22	61.92
IBO	23	47.25
ICM	23	59.5
ICJ	25	63.42
IDC	26	64.67
ICR	27	59.75
IBR	28	57.42
ICI	31	54.5
IBD	33	82.25
ICF	35	70.33
IDE	35	70.58
IBM	36	57.5
IBF	38	63.67
IBZ	38	44.17
IBY	41	48.92
ICV	43	58.42
IBX	46	70.5
IBK	53	72.25
IAZ	55	81.08
IBP	55	62.67
IBQ	57	81
ICK	57	69.67
ICE	66	72.83
ICS	68	85.92

Each row corresponds to a different listener whose subject code is given on the far left. All demographic information was provided by the listener in a survey. Age at onset of deafness was rounded to the nearest 6 month period. Age at testing is given in years.

#### Stimuli and testing apparatus

The stimuli and testing apparatus employed in this study were described previously by Jones and colleagues [27]. Stimuli were trains of four pink noise bursts of 170 ms duration separated by a 50-ms inter-stimulus interval. They were presented at an average level of 50 decibels sound pressure level, A-weighted [dB(A)]. This level was chosen to prevent the activation of automatic gain control of Cochlear devices. On each presentation, the level was roved by ±4 dB to discourage the use of monaural loudness cues. Similarly, ±10 dB spectral rove [53] was applied to discourage the use of monaural spectral cues due to room modes, or consistent differences in the spectral makeup of a signal that vary depending upon the reflections associated with different wall geometries that may affect localization [35, 54]. Signal processing and presentation were completed on a desktop computer using custom-written MATLAB software.

Stimuli were presented from 19 identical loudspeakers (Center/Surround IV; Cambridge SoundWorks) mounted in a semicircular, half-arc approximately 1.2 m from listeners’ heads. Speakers were spaced in 10-degree increments spanning ±90 degrees azimuth. The height of the seat for listeners was adjusted to zero degrees elevation with respect to loudspeakers. Loudspeakers were covered with a dark, acoustically transparent curtain. Stimuli were presented via a Tucker-Davis Technologies System3 with RP2.1, HB7, and PA5 units (digital processor, amplifier, and attenuator, respectively) with a 48-kHz sampling rate. All testing was completed in a single-walled, sound-attenuating booth (Industrial Acoustics Company, Inc.) with dimensions of 2.90 × 2.74 × 2.44 m. Sound-attenuating foam was attached to walls to reduce reflections in the booth.

#### Task

All testing was completed using listeners’ personal devices with everyday settings. Prior to testing, a pink noise burst was presented from a loudspeaker directly in front of the listener’s head. If the perceived location of the speaker was biased toward one side, the volume on the sound processor was adjusted until the listener perceived a centered image. Unfortunately, this information was not recorded during testing and we are therefore unable to report the listeners requiring loudness adjustments. Familiarization was completed prior to testing to familiarize listeners with the task by demonstrating how to use the graphical user interface and explaining the task to listeners. Listeners were instructed to face forward and keep their heads as still as possible before beginning each block of testing.

On each trial, stimuli were presented from one of the 19 loudspeakers. The task of the listener was to identify the location of the target loudspeaker. Listeners initiated each stimulus presentation using a graphical user interface implemented in MATLAB on a touchscreen located directly in front of them. Following stimulus presentation, listeners could respond on a visual representation of the semi-circular arc as to the perceived location of each sound. The response location appeared on the graphical user interface and could be adjusted until the listener was satisfied with their decision. The locations of loudspeakers were not shown in the visual representation and responses were restricted to ±90 degrees azimuth. Listeners were not able to repeat stimulus presentations. Once a listener made their decision, they would end the trial and a new trial would be initiated. No feedback was provided after each trial. Testing was completed in three blocks consisting of 95 trials, or five repetitions per loudspeaker. This resulted in a total of 285 trials total, or 15 repetitions per loudspeaker, and took approximately one hour to collect. Listeners with NH were only presented with 10 repetitions per loudspeaker.

#### Analyses

To address the goals of the present study, the data were analyzed with four different measures of localization performance. The first measure, RMS error, is by far the most common metric of localization performance in the NH and BiCI literature (e.g., [3, 12, 14, 26, 29, 32, 39, 55]). As mentioned in the Introduction, it characterizes average error and is insensitive to some differences in the pattern of localization responses. The second measure, LSI, was proposed as a statistical measure of sensitivity by assessing the pattern of localization responses [22]. The third measure, parameters of a sigmoidal curve fit to the target vs. response angles, characterizes the pattern of mean response angles, inspired by Jones and colleagues [27]. The final measure is a novel analysis approach developed for the purpose of this study. Listeners are sorted into meaningfully different categories based upon their pattern of localization responses when compared against bootstrapped simulations from each category. A brief description is provided in the Results section, and additional details are provided in the supplementary code and appendix that accompany this paper.

**Fig 2:**
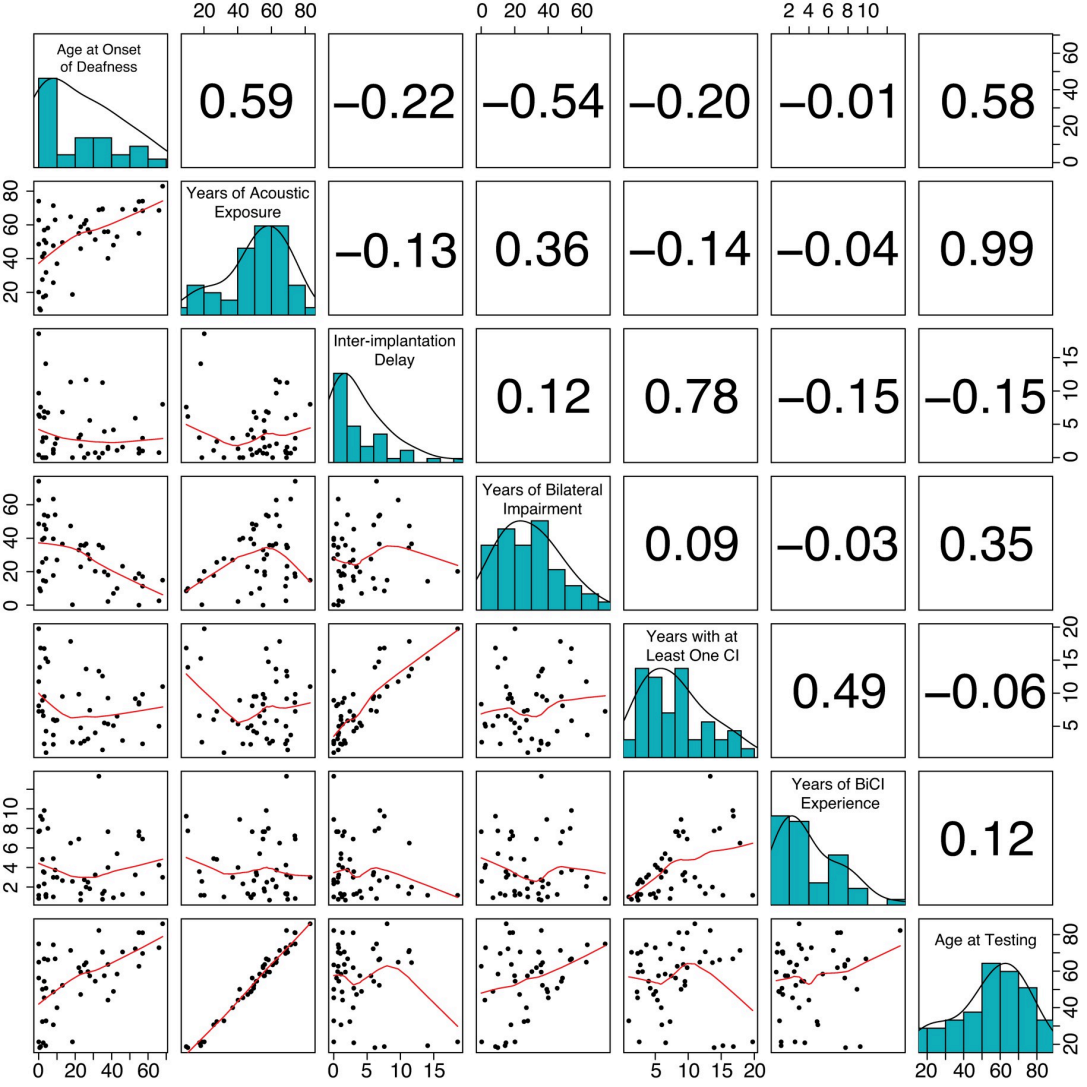
Relationship between patient-dependent factors. Each row and column correspond to a different patient-dependent factor that is given along the diagonal with a histogram showing its values. Values in each cell of the upper-triangle correspond to Pearson correlation coefficients. Plots in each cell of the lower-triangle show the relationship between two factors.

The patient-dependent factors considered in the present study are shown in Fig 2. Onset of deafness was determined by the answers to the questions: “Did you ever experience hearing? If so, for how long? When did you first start wearing a hearing aid in each ear?” Duration of bilateral impairment was computed as the number of years between age at onset of deafness and age at second implantation. The duration of acoustic exposure was calculated as the number of years between birth and age at second implantation. The inter-implantation delay was calculated as the number of years between age at first and second implantation. Years with bilateral and at least one CI were computed as the number of years from first or second implantation to age at testing, respectively. Together these factors capture information relevant to a listener’s early and later auditory deprivation and experience. For additional details, please see [4]. To determine which factors to include in regression models associated with the different measures of performance, we used the *step* function in the lmerTest package for mixed-effects models and stats package for fixed-effects models in R. This performs stepwise model comparison to determine the model with the smallest Akaike information criterion (i.e., a measure of predictive power similar to adjusted R^2^ in regression), removing factors that provide the least prediction and balancing the number of parameters with the increased model fit. It is compatible with fixed- and mixed-effects models. As seen in Fig 2, some of these factors are highly related to one another. We chose to exclude factors with a high effect size correlation (> .75) from the same model selection process. Thus, only years of bilateral hearing impairment or age at testing were included within the same stepwise model comparison.

## Results

### Individual results

**Fig 3:**
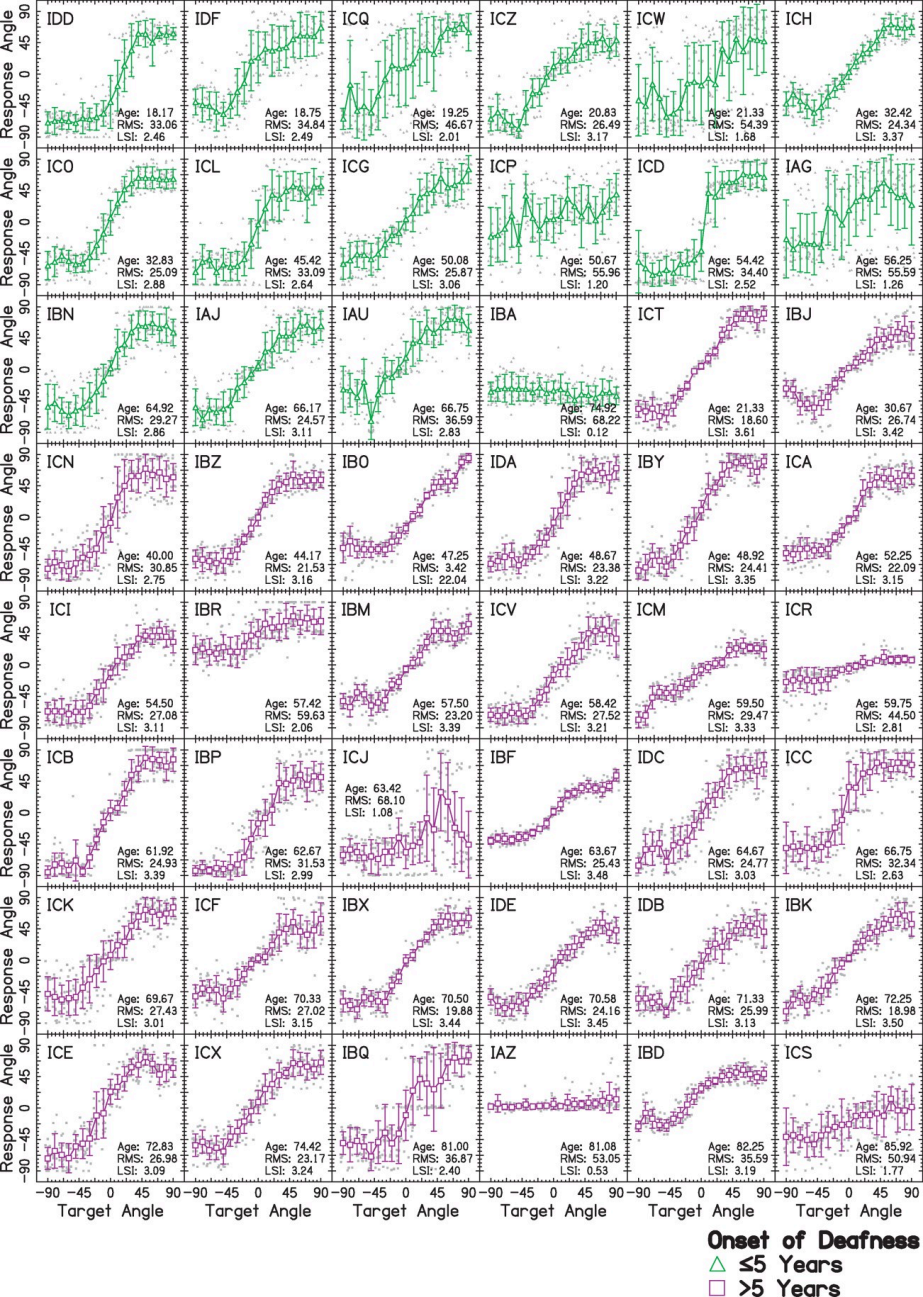
Sound source localization for forty-eight listeners with BiCIs. The x-axis shows the angle from which the target speaker presented pink noise bursts. The y-axis shows the angle from which the listener perceived the sound to be presented. Each panel shows a different listener whose subject codes are given in the top-left corner. Mean ±1 standard deviation for listeners who experienced an onset of deafness either ≤5 years or >5 years are shown with green △ and magenta ▢ symbols, respectively. Individual responses are shown jittered in gray in the background. Within each group, listeners are arranged by increasing age at testing, which is given in years as “Age” in years within each panel. Additionally, the RMS error over all target angles and LSI are listed in the bottom-right of each panel.

Results for each listener with BiCIs are plotted in Fig 3. Listeners were classified into two groups corresponding to their self-reported age at onset of deafness. This classification into two categories was provided for two reasons. Firstly, it is thought that there is a critical period during which the auditory brainstem organizes itself to represent spatial cues [6, 51]. Research in children with BiCIs suggests that localization performance becomes considerably worse if deafness occurs in early childhood, before approximately five years of age [7], as does sensitivity to ITDs [56, 57]. Listeners with BiCIs are suspected to rely on ILDs for localization [33, 41, 58], and ITD and ILD thresholds are predictive of one another [4]. Secondly, by including age at onset of deafness as a categorical variable instead of a continuous one, effects of multicollinearity (relationships between patient-dependent factors that explain the same trends in the dependent variable) are accounted for. The present study’s relatively large dataset was collected across many patients, making it ideal to explore the implications of patient-dependent factors on localization performance.

### Localization errors and response patterns

#### Root-mean-square error

**Fig 4:**
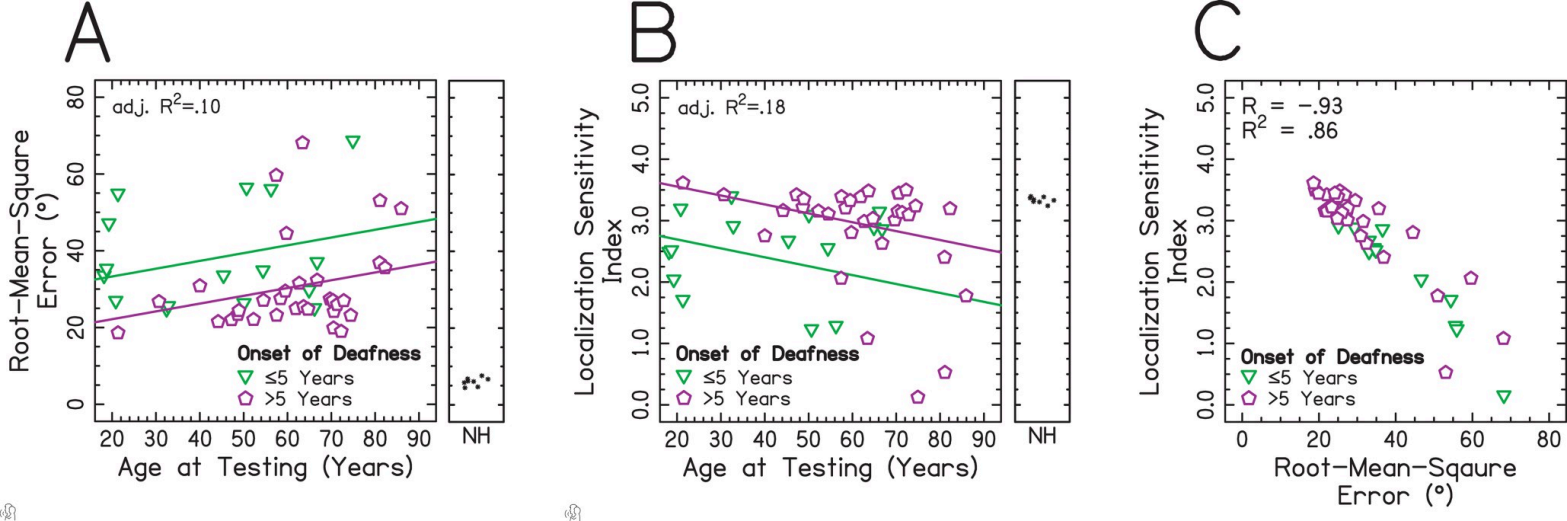
Relationship between RMS error over all target angles, the LSI, and patient-dependent factors. Individuals with an onset of deafness ≤5 or >5 years are shown with green ▽ and magenta ⬠ symbols, respectively. (A) The x-axis represents age at testing in years. The y-axis represents the RMS error, where higher values indicate poorer performance. The adjusted R^2^ was taken from a regression including factors of onset of deafness group and age at testing. The sub-panel offset to the right shows data from listeners with NH. (B) The x-axis represents the age at testing in years. The y-axis represents the LSI, where lower values indicate poorer performance. The adjusted R^2^ was taken from a regression including factors of onset of deafness group and age at testing. The sub-panel offset to the right shows data from NH listeners. (C) The x- and y-axes represent the LSI and RMS error, respectively. The R^2^ corresponds to the coefficient of determination taken from a correlation between overall RMS error and the LSI.

The relationship between patient-dependent factors and RMS error was assessed over all target angles and is shown in Fig 4A. These factors were chosen based upon the results of the stepwise model comparison test. The RMS error tended to increase with increasing age at testing, but there was considerable variability in this effect. Data in Fig 4A were fitted with a multiple linear regression with dependent variable RMS error and fixed-effects of age of onset of deafness (categories of ≤5 and >5 years) and age at testing (continuous). The multiple regression significantly predicted RMS error (F(2,45) = 3.682, p < .05), providing a relatively poor fit to the data with an adjusted R^2^ = 0.10. Age at onset of deafness had a significant effect (t(45) = -2.616, p < .05), with an average of 11.132 degrees less RMS error for listeners with a later age at onset of deafness. There was no significant effect of age at testing (t(45) = 1.856, p = .070).

An alternative analysis approach would be to evaluate the RMS error for each target angle. This would account for, to some degree, differences in the amount of error across target angles. As discussed in previous reports [3, 13, 27] and shown in Fig 3, the amount of error tends to increase at lateral target angles for listeners with BiCIs. One previous approach to address this problem has been to only take the RMS error over a limited range of target locations (e.g., ±60 degrees [13]). However, this approach ignores valuable data and removes important features from the pattern of localization responses. Thus, mixed-effects regression was implemented in the lme4 package [59] in version 3.5.1 of R [60], and included the following fixed-effects: square of target angle, age at onset of deafness (categories of ≤5 and >5 years), and their interaction. This model was chosen based on the results of a stepwise model comparison test. The square of target angle was included in the analysis to capture the fact that the amount of error grew at a faster rate between lateral target angles than more medial angles. Intercept was treated as a random effect and allowed to vary randomly with listener. Including intercept as a random effect accounts for differences in average performance across individuals. To avoid violating assumptions in the regression (linear relationship between independent variables with dependent variable, constant variance, and normality of residuals), the RMS error was transformed by taking the natural logarithm. The log-transformed RMS error had a minimum of 1.457, median of 3.227, mean of 3.253, and maximum of 4.965. Degrees of freedom were calculated using the Satterwhaite approximation [61].

The results of the stepwise model comparison regression analysis are given in Table 2. There were significant effects of age at onset of deafness and squared target angle. The point estimates for both effects were in the anticipated direction. That is, an earlier onset of deafness and further lateral target angle were both associated with increased RMS error.

**Table 2:** Fixed-effects coefficients from mixed-effects model.

Effect	Estimate	Standard Error	Degrees of Freedom	t-statistic	p
Intercept	3.339	0.082	61	40.489	< .0001
Onset of Deafness (OD)	-0.401	0.101	61	-3.971	< .001
Sq. Target Angle (STA)	3.626×10^−5^	9.210×10^−6^	864	3.937	< .0001
OD × STA	3.668×10^−5^	1.128×10^−5^	864	3.252	< .01

The factors in the table were used to predict log(RMS error) with a mixed-effects linear regression including random intercepts associated with each listener and the fixed-effect presented in this table.

There was a significant age of onset of deafness × squared target angle interaction. Surprisingly, this interaction had a positive coefficient. This suggests that the amount of error at further lateral target angles was smaller, proportionally, for earlier onsets of deafness. Squared target angle varied between 0 and 8100. Thus, at the lateral-most target angles, log(RMS error) increased by 0.239 for the earlier onset of deafness group and 0.591 for the later onset of deafness group. Thus, for listeners with a later onset of deafness, the average error associated with increasingly lateral target angles would have been more than double that of listeners with an earlier onset of deafness. This suggests that listeners with an earlier onset of deafness had greater average error and proportionally fewer errors near the center of the head compared to listeners with a later onset of deafness. The resulting error at the most lateral target angles was similar between the earlier and later onset of deafness groups, suggesting that the difference between groups was driven primarily by errors for medial target angles. The implications of this interaction are considered further in the Discussion.

#### Localization sensitivity analysis

The LSI was formulated by Zheng and colleagues [22] with the intention of providing a statistic that revealed information about the pattern of localization responses and listeners’ localization acuity. The LSI is a summary statistic that is similar in spirit to generating a confusion matrix and averaging the confusions for all target and response angles. The rows in a confusion matrix correspond to the dependent variable (i.e., response angle) and the columns correspond to the independent variable (i.e., target angle). The value of each element in the matrix is determined by the number of occurrences (cases where the response angle equaled a particular target angle). Because the dependent variable of the localization task used in the present study (i.e., response angle) is continuously distributed, it is not possible to present the data in a confusion matrix. Instead, the *distribution of responses* was compared for each pair of target angles, resulting in a matrix of values that indicated their degree of similarity. The LSI was calculated by computing the Kruskall-Wallis (i.e., rank order-based) statistic between the responses at two different target angles, calculating the p-value of said statistic, converting the p-value to the corresponding positive z-score, and averaging z-scores across all pairs of target angles. Thus, the LSI is akin to the mean d’ [62] across all pairs of target angles. For reference, calculation of d’ is completed by taking the difference between means of two different distributions assumed to be standard normal. In this case, the distributions being compared are response angles for two different target angle presentations (i.e., for all pair-wise combinations). Since d’ is in z-score units, the LSI has a similar interpretation to the average of many d’ values. When the LSI is near zero, this indicates poor performance (overlapping response angles for differing target angles). When the LSI is large, this indicates optimal performance (disparate response angles for differing target angles). For additional details, see the paper in which the LSI was formulated [22].

The relationship between the LSI and patient-dependent factors is shown in Fig 4B. These factors were chosen based upon the results of the stepwise model comparison test. The LSI tended to decrease with increasing age at testing, though this effect was quite variable across participants. Data in Fig 4B were fitted with a multiple linear regression with dependent variable LSI and fixed-effects of age of onset of deafness (categories of ≤5 and >5 years) and age at testing (continuous). As is visually apparent in Fig 4B, the multiple regression was significantly predictive of the LSI (F(2,45) = 6.022, p < .01) and provided a relatively poor fit to the data yielding an adjusted R^2^ = .18. Age at onset of deafness had a significant effect (t(45) = 3.393, p < .01), with an average of 0.861 greater LSI for listeners with a later age at onset of deafness. There was also a significant effect of age at testing (t(45) = -2.225, p < .05), with each increasing year resulting in approximately 0.015 units less localization sensitivity. For the oldest listener, this resulted in 1.289 units less localization sensitivity. Because age at testing and years of acoustic hearing were so strongly correlated, their relationship will be discussed in more detail in the Discussion.

The results showed that the LSI had an inverse relationship with RMS error across all target angles (Fig 4C). A Pearson correlation revealed a strong correlation between the LSI and RMS error (R^2^ = .86). This is consistent with the original paper including LSI’s formulation for localization with children who use BiCIs [22]. Fig 4C suggests that for many values of the RMS error (x-axis), especially until roughly 40 degrees of RMS error where there is greater residual error in the correlation, the LSI (y-axis) provides the same information for localization performance. This is intuitive, since for low values of the RMS error, the LSI would be high in general. When the RMS error is high, however, the types of errors made by participants may differ, suggesting that it might be more useful to determine the types of errors made by listeners. This suggests that LSI is providing new and important information that is not provided by overall RMS error at the largest values of overall RMS error, which provides greater insight into its utility assessing sound source localization development in children. The issue with the LSI is that it is only indicative of a greater amount of confusions. Therefore, the LSI does not give a meaningful indication as to the pattern of localization responses. Moreover, there are examples where the LSI is similar between listeners but the pattern of localization responses is quite different. One example of such a case is presented in Fig 1. Listener IAG (whose LSI is 1.26), biased toward medial angles, readily confuses the loudspeaker at zero degrees with ±30 degrees, as does listener IAZ (whose LSI is 1.33), who does not exhibit this bias. However, as is obvious from Fig 1, the localization response patterns of listeners IAG and IAZ are extremely different from one another. Thus, the LSI does not appear to be useful for the current experimental goals but may prove useful for other applications. This is considered further in the Discussion.

#### Four-parameter logistic functions

It may also be of interest to determine how a patient with BiCIs performs on a localization task by describing the shape of the function. One approach used by Jones and colleagues [27] was to fit the error across absolute target angle (generalized between the left and right) with orthogonal polynomials. This approach collapses errors across both sides of the head. The issue with collapsing errors across both sides of the head is that listeners with BiCIs can exhibit different kinds of errors on either side of the head depending on the listener, CI configurations (e.g., unilateral vs. bilateral), and stimulus [3, 13, 31, 40, 42]. Instead, the data in the present study were fit with an S-shaped function that models the maximum and minimum response angles as well as the slope. These parameters describe important features of localization performance and give some indication of where a listener struggles to identify sound sources. Clearly there is a relationship between the maximum, minimum, and slope of the logistic function, but they do not always co-vary. For example, ideal performance would result in a high maximum and low minimum, with a 1:1 slope between target and response angle. However, a person who is insensitive to small changes in location and is able to accurately judge left from right may still identify a sound far to the left or right, but transition between these extremes as soon as a sound crosses the center of the head. This strategy has been noted in children with BiCIs [22]. Separately, the maximum and minimum response angles also provide indices of bias toward one side of the head.

In the present study, we chose to fit data using the four-parameter logistic function shown in Eq 1:

$$y_{ij}=\alpha +\frac{\beta -\alpha }{S(\frac{\mu -x_{ij}}{\sigma })}+\epsilon_{ij}$$ (1)

where *y*_*ij*_ represents the *i*th response for the *j*th target angle, *α* represents the right-most response angle, *β* represents the left-most response angle, *S(x)* represents the sigmoidal function *S(x) = 1/(1+exp(-x))*, *μ* represents the center of the curve along the target angles, *x*_*ij*_ represents the *i*th repetition at the *j*th target angle, *σ* represents the slope of the curve, and *ε*_ij_ represents one instance (for the *i*th repetition at the *j*th target angle) of random errors that are independently and identically distributed.

**Fig 5:**
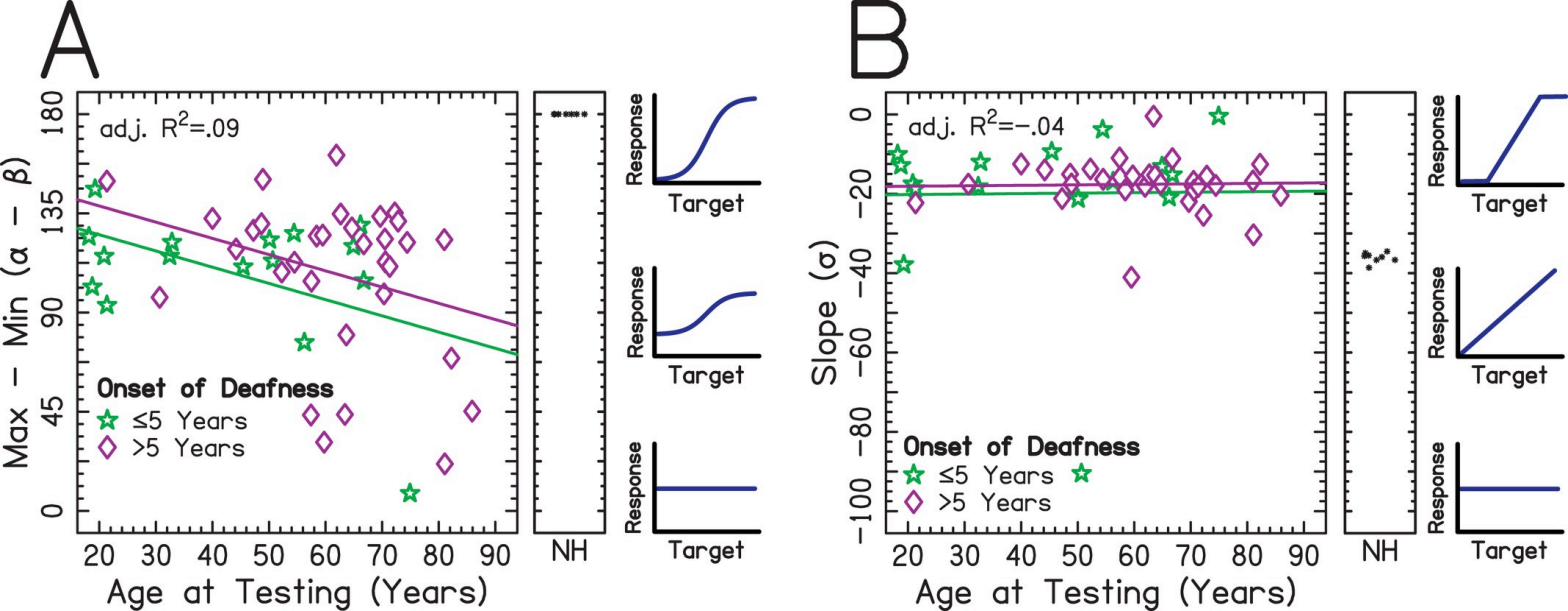
Relationship between logistic parameters and patient-dependent factors. Individuals with an onset of deafness ≤5 or >5 years are shown in green ☆ and magenta ◊ symbols, respectively. The x-axis represents age at testing in years. The adjusted R^2^ was taken from a regression including factors of onset of deafness group and age at testing. The sub-panel offset to the right shows data from NH listeners. An illustration of the localization function is shown to the right of the sub-panels for aid of interpretation. (A) The y-axis represents the range of response angles (*α*–*β* from Eq 1) of the localization function, where lower values indicate smaller ranges of responses and poorer performance. (B) The y-axis represents the slope (*σ* from Eq 1) of the localization function.

In general, this modeling problem requires at least three, and typically four, parameters. Four-parameter logistic functions have practically interpretable parameters corresponding to the upper- and lower-asymptote, midpoint along the x-axis, and slope (see Eq 1). Parameter estimates were generated by fitting functions to localization data iteratively with non-linear least squares estimation using the Curve Fitting Toolbox of MATLAB and the trust-region-reflective algorithm [63] to update parameter estimates and minimize residual error. Estimates of *β* and *α* were bounded between [–90, 90] and were initialized at -90 and 90, respectively. Estimates of *μ* were bounded between [–90, 90] and were initialized at 0. Estimates of *σ* were bounded between [–100, 100] and were initialized at -50, corresponding to a nearly 1:1 relationship between target and response angles. The relationship between parameter estimates and patient-dependent factors is illustrated in Fig 5.

We chose to evaluate two important features of the localization function: the range over which responses fell (*α*–*β* in Eq 1; Fig 5A) and the slope (*σ* in Eq 1; Fig 5B). Values nearer to 180 in the range of responses for the localization function are indicative of good performance, showing that listeners perceived far-lateral locations when targets were presented there. This is similar to studies of the perceived intracranial locations of sounds when interaural cues are manipulated and presented via headphones or direct stimulation that evaluate range of responses [64–67]. Since many of the errors in localization performance occur at far lateral target angles, the range of responses, along with the slope, suggests the range of target angles for which listeners are sensitive to changes in location. From Fig 5, it is visually apparent that there is little or no relationship between the patient-dependent factors explored for the listeners in this study and the features of the localization function. We chose to include the same predictors as those used for overall RMS and LSI for the sake of consistency. Additional stepwise model selections revealed that no regression models provided significant predictions of the data. While listeners with BiCIs varied somewhat in the parameters associated with the logistic function, listeners with NH showed remarkably similar parameter estimates. The range over which responses fell was highly correlated with RMS error (Pearson *r* = -0.732) and LSI (Pearson *r* = 0.661). In contrast, slope was not correlated with RMS error (Pearson *r* = -0.081) or LSI (Pearson *r* = 0.109).

#### Unsupervised machine learning

While fitting the data using a logistic curve has the advantage that the function can be described using combinations of four parameters, this method of analysis ignores the variability across target loudspeaker locations. Further, as described by Zheng and colleagues [22] and Grieco-Calub and Litovsky [10], often times it is more intuitive to interpret localization performance in terms of “categories.” A similar approach has been used for distinguishing psychometric functions from different groups of listeners in pitch perception experiments [68]. That is, there are meaningful characteristics by which listeners with BiCIs differ from one another. The issue is that there is not a standard analysis technique to address this problem. The solution proposed by Grieco-Calub and Litovsky [10] relies on comparison of specific features of collected data against a control group. The solution proposed by Reiss and colleagues [68] relies on significant changes in parameters of functions depending upon which ear(s) are stimulated within the same individuals, which does not apply directly to the present study, but could be employed in, for example, a longitudinal study. Both studies share the approach of comparing data from a particular condition or group against some baseline.

**Fig 6:**
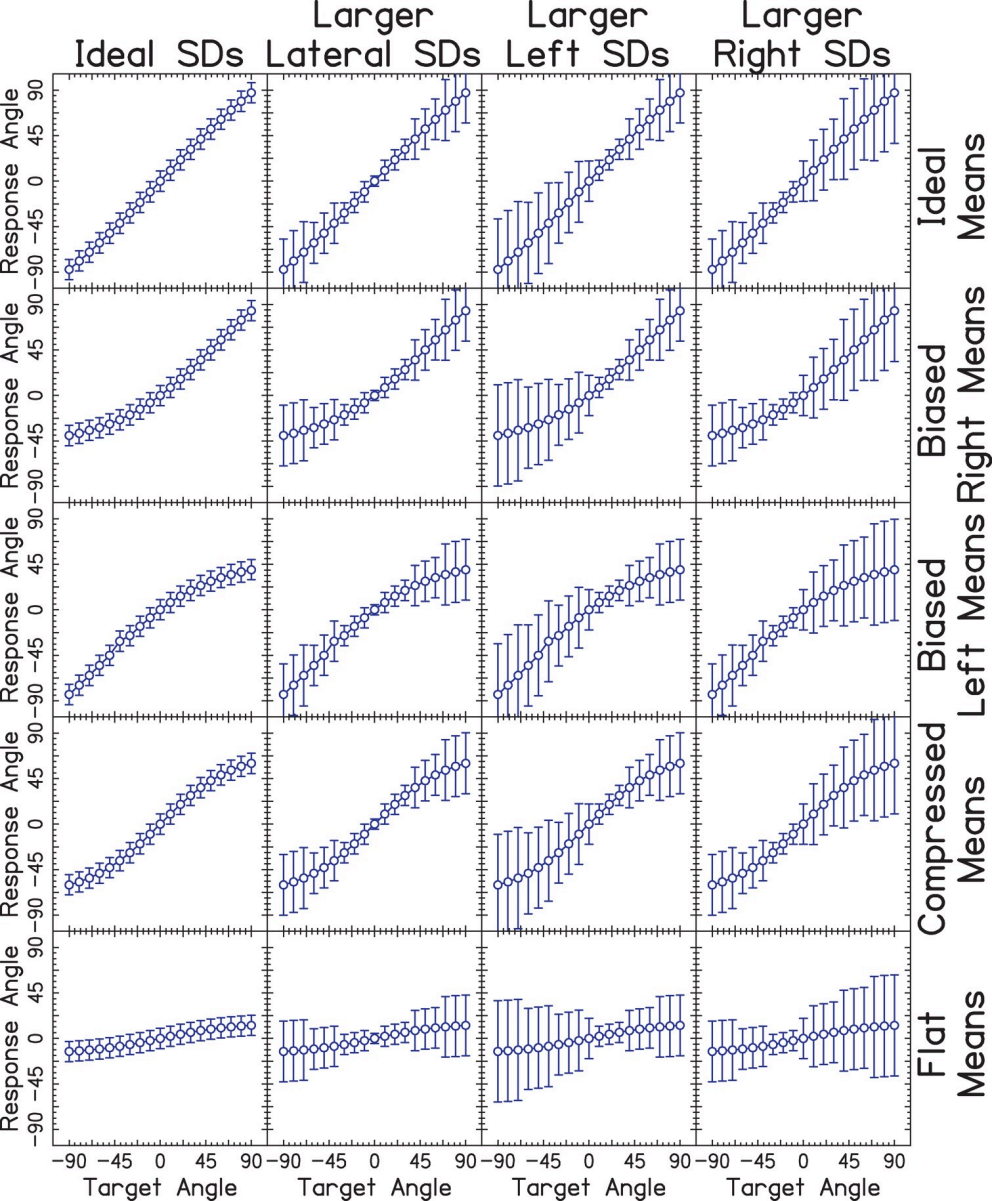
Categories of localization performance. Plotted as in Fig 3, except that each panel corresponds to a different category of localization performance as defined by experimenters. Each row and column represent a different distribution of mean and standard deviation (SD) of response angles, respectively.

To address this problem, we developed a new, more adaptable method of analysis inspired by those used by Zheng and colleagues [22] and Grieco-Calub and Litovsky [10], in which listeners were sorted into predefined categories according to their similarity to data simulated by bootstrapping from these categories. While we defined the categories in the present study, the method is flexible such that parameters of simulated data could be adapted by future researchers and refined by the field. Simulated sampling of these categories was completed using bootstrapping, and data from listeners were then compared against bootstrapped samples. An illustration of the different categories of responses is shown in Fig 6.

The choice of parameters and corresponding categories provided in the present study were guided by Zheng et al. [22]. They described five broad categories of localization functions, ranging from NH-like means and small standard deviations to a nearly random distribution of responses across target angles (i.e., flat means and large standard deviations). Different patterns of means and standard deviations are intended to reflect everyday-relevant changes in localization performance important for device design, changes across time, and, as studied here, differences between groups of patients. We consider the pattern across means to reflect the bias (or lack thereof) in the listeners’ responses, and the pattern across standard deviation to reflect reliability (variability) of their spatial perception across presentation intervals.

Response angle means and SDs at each target angle from each listener with BiCIs were compared against 50 simulated subjects of each category from Fig 6. Each of these simulated subjects had 15 repetitions per target angle, i.e., the same as listeners in the experiment, and were classified into one of twenty “clusters” using the partitioning around medoids (PAM) unsupervised machine learning algorithm [69]. This resulted in a total of 1000 simulated subjects (50 simulated subjects from 20 categories) compared against one listener with BiCIs. The category of the simulated localization data could be extracted following clustering. Accordingly, the category of the listener with BiCIs was taken as the mode of the simulated categories within the same cluster. For example, say that listener IAG was sorted into a cluster containing the following: 36 Biased Right Means, Larger Right SDs, 8 Compressed Means, Larger Right SDs, and 7 Biased Left Means, Larger Left SDs from Fig 6. Then the category of listener IAG would be taken as Biased Right Means, Larger Left SDs because this was the mode of the cluster into which listener IAG was categorized. This process was repeated 50 times with newly simulated data for each listener so that the reliability of clustering could be assessed.

**Fig 7:**
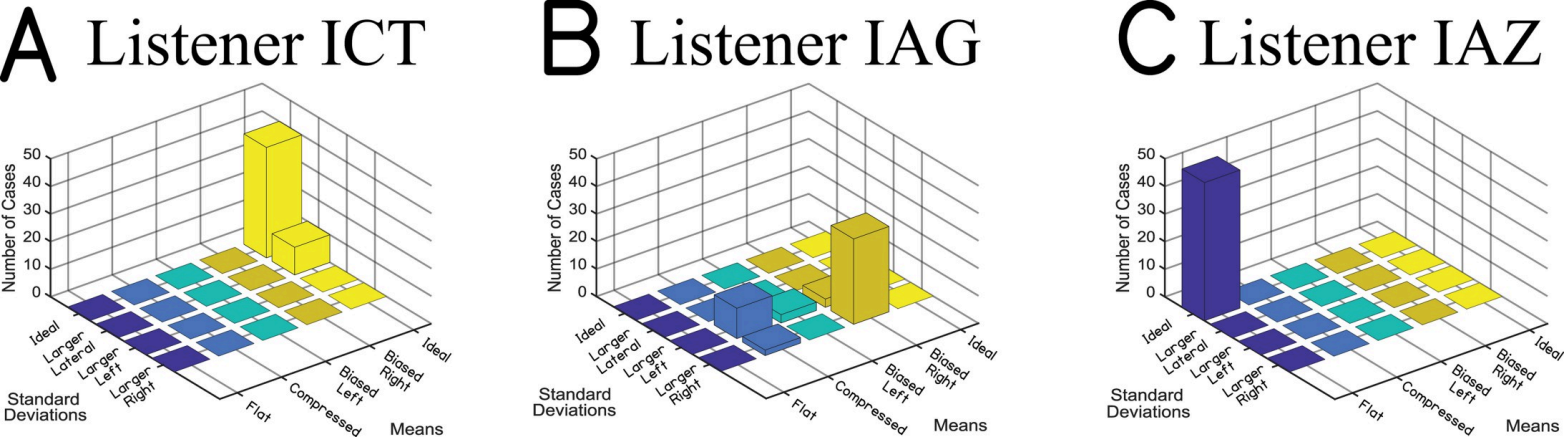
Examples of frequency of assignment to specific categories defined in Fig 6. (A), (B), and (C) correspond to listeners ICT, IAG, and IAZ, respectively. The x-axis corresponds to the mean category, the y-axis corresponds to the SD category, and the z-axis corresponds to the frequency of assignment. Since this process was repeated 50 times, there were a total of 50 assignments in each subplot.

The method described above uses bootstrapping (many simulations from a series of localization patterns described in Fig 6) to sort listeners into categories. Bootstrapping is essential to our approach since each localization function from the data observed from a listener with BiCIs is only a sample from their underlying localization category. That is, data are sampled from some population of localization responses that would be produced by a particular listener in one particular experimental setup. These bootstrap simulations are generated iteratively, and the process is repeated 50 times to ensure reliability of the category assignment for each listener with BiCIs. Thus, the method returns a single result as well as the reliability with which a listener was sorted into a particular category. Reliability of clustering for the same three listeners from Fig 1 is shown in Fig 7. In these examples, each panel corresponds to the same categories of performance provided in Fig 6. The numbers and colors correspond to the frequency with which each listener was sorted into each category. For listener ICT, the PAM algorithm most consistently sorted their localization responses with the Ideal Means, Ideal SDs category, and sometimes into the Ideal Means, Larger Lateral SDs category. For listener IAG, the algorithm was less consistent overall. For listener IAZ, however, the algorithm provides unanimous support for the Flat Means, Ideal SDs category. Where both RMS error and the LSI failed to distinguish between these listeners, this new method of analysis using bootstrap simulations and unsupervised machine learning succeeded. Further, the output of the algorithm can be used to determine the confidence of a particular category assignment.

**Fig 8:**
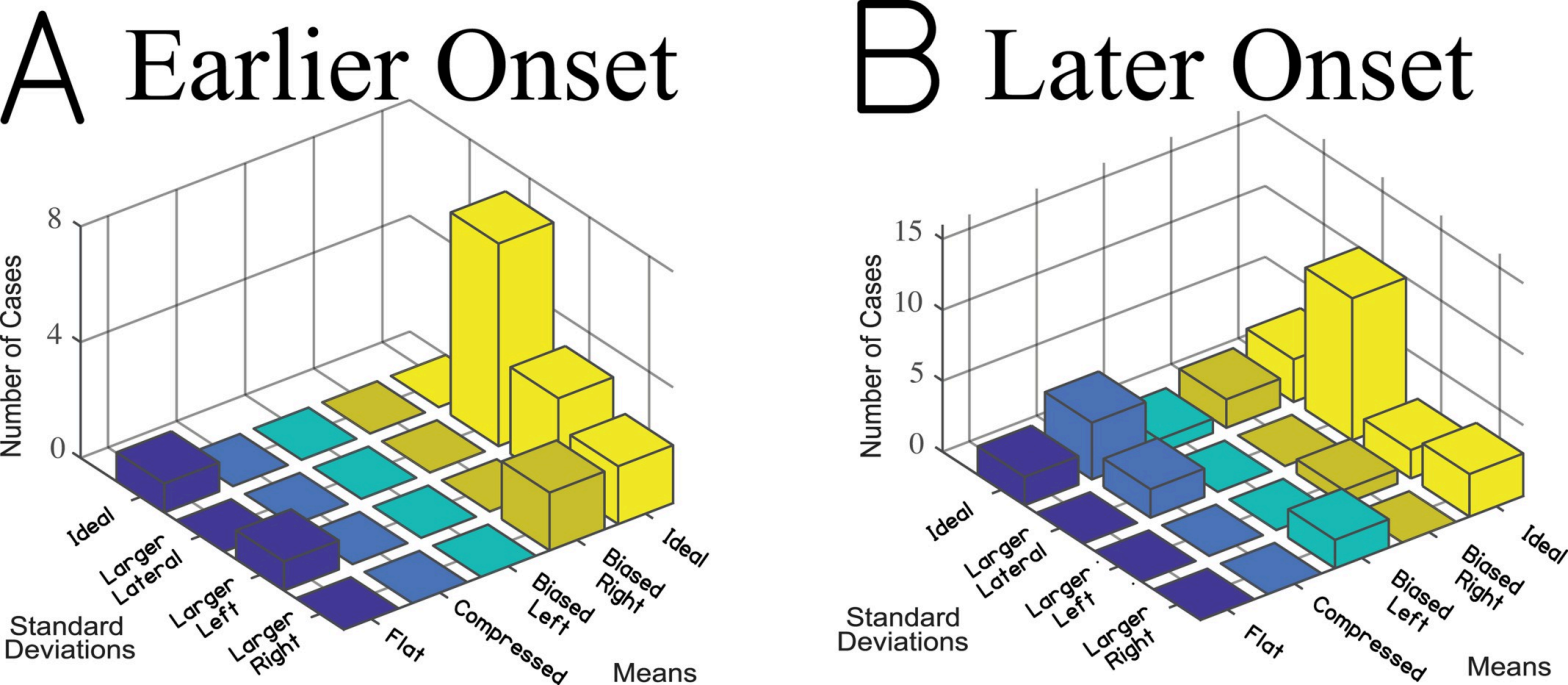
Histogram indicating category assignments by onset of deafness group. The x-axis corresponds to the mean category, the y-axis corresponds to the SD category, and the z-axis corresponds to the frequency of assignment (with a total of 16 listeners in panel A and 32 listeners in panel B). (A) and (B) correspond to the ≤5 and >5 years onset of deafness groups, respectively. The range of the z-axis was scaled based upon the number of listeners in each group, with the maximum equal to half of the group size.

The ultimate category assignment was determined by taking the mode of results like those presented Fig 7 for every listener. A histogram of category assignments is plotted in Fig 8 across all 48 listeners in the study, separated into two separate panels corresponding to the earlier and later onset of deafness groups. We chose to include age at onset of deafness as the predictor because it was the most robust predictor with the other measures provided in the present manuscript. This new method of analysis revealed that the later onset of deafness group (Fig 8B) was more likely to be sorted into the ideal SDs categories than the earlier onset of deafness group. A chi-square test for difference in counts indicated that there were significantly more listeners sorted into the ideal SDs categories for the later (12 ideal SDs, 20 other; a rate of 3:5) compared to earlier (1 ideal SDs, 15 other; a rate of 1:15) onset of deafness groups (χ^2^(1) = 5.275, p < .05). This result is consistent with the hypothesis that an earlier onset of deafness leads to poorer localization performance; specifically, less consistent perceptions of sound location from one interval to the next. A power analysis assuming an equal sample size to that obtained in the present study based on 10,000 simulations in R and an alpha level of .05 revealed power of .70. In other words, based on the result obtained, one would expect to commit a Type II error (failure to reject the null hypothesis) approximately 30% of the time.

Additionally, it seemed that there might be a difference between the assignment to the ideal means category between the listeners with earlier and later onset of deafness, with earlier (12 ideal means, 4 other; a rate of 3:1) compared to later (18 ideal means, 14 other; a rate of 9:7) onset of deafness patients showing more listeners in the ideal means categories, contrary to our initial hypothesis. However, a chi-square test for difference in counts indicated that there was not sufficient statistical evidence to conclude that the number of listeners in the ideal means categories differed between onset of deafness groups (χ^2^(1) = 1.600, p = .206). No continuity corrections were completed with either chi-square test. For comparison, data from listeners with NH were also tested. All iterations for each of the eight participants were sorted into the ideal means, ideal SDs category.

Referring back to Fig 3, the greater amounts of variability at each target angle for the earlier (1 ideal SDs, 15 other; a rate of 1:15) compared to the later (12 ideal SDs, 20 other; a rate of 3:5) onset of deafness group which significantly distinguished them from the later onset of deafness group can easily be seen, though it was not obvious initially. Thus, this new method of analysis was sensitive to differences in localization functions and showed a new trend in the data. It should also be noted that there was considerable heterogeneity in the mean response angles in both groups. While the difference between groups was not significant in the present study, these differences may be explained by other patient-dependent factors that were not explored.

## Discussion

The goal of the present study was to determine whether patient-dependent factors related to auditory deprivation, experience with CIs, and age were predictive of the amount of error and pattern of localization responses in listeners with BiCIs. It was hypothesized that earlier onsets of deafness would lead to more errors because of detrimental changes to encoding and decoding of binaural cues. Specifically, it was predicted that an earlier onset of deafness would be characterized by mean localization responses that were biased and exhibited large amounts of variability. It was further hypothesized that longer periods of auditory deprivation, shorter periods of experience with BiCIs, and older age at testing would be associated with larger amounts of variability. The results from the present experiment were consistent with our predictions, and showed that age at onset of deafness and age at testing were the most predictive of performance. The mixed-effects regression provided evidence that listeners who experienced an early onset of deafness (≤5 years) tended to make greater errors at lateral target angles, and proportionally more errors at medial target angles, compared to listeners with a later onset of deafness. This implies that earlier onset of deafness lead to more errors in localization performance for listeners with BiCIs, especially at medial target angles. The novel machine learning analysis revealed that listeners with an earlier onset of deafness were more likely to exhibit non-ideal standard deviations of lateralization responses, which likely drove the mixed-effects regression results. This suggests that the responses of listeners with earlier onset of deafness were less reliable from trial to trial. Specific results are discussed in greater context below.

### Factors that limit binaural hearing outcomes

Auditory deprivation has different effects depending upon when it occurs during the lifespan. There is evidence of activity-dependent critical periods for the formation of circuits used in binaural computations and changes in the auditory system with older age and deafness, both of which contribute uniquely to auditory anatomy and physiology [6, 18, 51]. First, we outline the brainstem circuit responsible for encoding binaural cues. Then, we discuss how this circuit may change with auditory deprivation earlier or later in life and how this is expected to affect localization performance. Finally, we discuss what other factors might play a role in differences in localization performance across listeners, interacting with patient-dependent factors related to auditory deprivation studied here.

#### Review of binaural processing

After a sound is transmitted to the auditory nerve (via electrical stimulation for listeners with CIs or depolarization by hair cells for listeners with acoustic hearing), it first arrives at the cochlear nucleus. Bushy cells in the cochlear nucleus refine the temporal precision by integrating many auditory nerve inputs [70] and receiving inhibition from neighboring cells [71, 72]. This refined temporal precision is important for accurate encoding of ITDs, resulting in humans’ sensitivity on the order of microseconds, far below the refractory period for most neurons in the central nervous system. The medial superior olive (MSO) is suspected to code for fine-structure ITDs [73, 74]. It receives excitatory input from spherical bushy cells on both sides of the brainstem [75, 76] and acts as a coincidence detector, exhibiting selective ITD-tuning. The MSO also receives inhibitory inputs from the lateral and medial nuclei of the trapezoid body [77], which plays a role in ITD tuning [78]. The lateral superior olive (LSO) is suspected to code for envelope ITDs [79, 80] and ILDs [81, 82]. It receives excitatory input from the ipsilateral cochlear nucleus and inhibitory inputs from the contralateral medial nucleus of the trapezoid body [83], which in turn receives its inputs from globular bushy cells [84]. There is ongoing debate as to whether the ipsilateral input to the LSO comes from spherical bushy cells [75] or T-stellate cells [85], both of which are found in the cochlear nucleus. In all three of these nuclei (the cochlear nucleus, MSO, and LSO), precisely timed excitation and inhibition are essential for the accurate encoding of binaural cues. Moreover, all three nuclei have demonstrated the presence of specialized inhibitory (hyperpolarization) channels that allow for their extraordinarily precise temporal properties [86]. Thus, changes in the strength of inputs (excitatory or inhibitory) and anatomical organization of these nuclei could be detrimental for binaural processing.

#### Deafness during development

Hearing loss during development leads to an imbalance in the ratio of excitation and inhibition in the cochlear nucleus, MSO, and LSO. The “central gain hypothesis” posits that hearing loss leads to an overall increase in excitation to compensate for the loss of input to the auditory system [87]. This conceptual model appears to provide a better explanation of physiological responses of the auditory cortex, but there is still compelling evidence in the brainstem and midbrain to suggest a shift in the balance of inhibition and excitation that accompanies hearing loss (e.g., [18, 51, 88]). Crucially, a shift in the balance of excitation and inhibition would affect the ability to encode binaural cues in the mature auditory system since the cochlear nucleus, MSO, and LSO rely on inhibition to maintain their exquisite temporal precision.

The results from the present study showed that localization responses were less reliable for listeners with earlier onset of deafness, driven primarily by medial target angles and greater amounts of variability. This is consistent with the notion that binaural encoding is less reliable in listeners with an earlier onset of deafness, but is inconsistent with the idea that responses are generally more biased. In fact, there was a non-significant trend in the unsupervised machine learning analyses to suggest that more listeners with an earlier onset of deafness tended to exhibit a higher proportion of ideal response angle means. This suggests that the primary limitation to localization performance is poorer binaural encoding, or that if the problem is with decoding, it relates to a listeners confidence or reliability in responses. Proportionally greater errors at lateral target angles for listeners with a later onset of deafness may suggest challenges using the greatest magnitude ITD or ILD provided by clinical processors in listeners with histories of acoustic hearing. It may be that the way ITDs or ILDs are represented by clinical processors is inconsistent with the spatial cues represented by acoustic hearing.

Results from the present study are generally consistent with many physiological studies in animals implying the presence of a critical period for the organization of the auditory brainstem. An imbalance of excitation and inhibition during early stages of development can cause malformation of anatomical structures in the brainstem [18, 51]. A developmental window during which the underlying cellular components that contribute to this anatomical reorganization are downregulated would make the binaural system susceptible to disorganization during early hearing loss [89]. Cells in the cochlear nucleus that project to binaural cells undergo morphological changes during development and are affected by hearing loss [90]. The post-synaptic density of the endbulb of Held synapse (i.e., specialized synapse in the cochlear nucleus) is preserved with early CI stimulation, but other morphological changes occur with deafness in cats despite stimulation [91]. In the MSO, Mongolian gerbils that were reared with omnidirectional noise show poorer tuning to ITDs compared to gerbils that were reared normally [92]. In the inferior colliculus, which receives direct projections from the MSO and LSO, rabbits reared with early hearing loss that receive BiCIs after maturity exhibit poorer tuning to ITDs and greater variability in cellular responses than animals that were reared normally or received BiCIs shortly after birth [88, 93]. When animals are raised normally and not deafened prior to CI stimulation, acoustical and CI stimulation can yield similar cellular responses [94], suggesting that deafness and device limitations are the primary contributors to little binaural sensitivity. Histological analysis demonstrated that early- and late-deafened animals showed similar deterioration of the auditory periphery [88], suggesting that reorganization in the brainstem and peripheral degradations contribute to changes in sensitivity to binaural cues. Behaviorally in humans, limited exposure to binaural hearing has also been associated with little or no sensitivity to ITDs [56, 57] and poorer localization outcomes [7].

In contrast, three studies in rats have shown a similar sensitivity in tuning between animals with NH and those who were early-deafened and received CI stimulation [95–97]. Supportive of their assertion that tuning was similar, many neurons recorded from the inferior colliculus in these rats were significantly tuned to ITDs, in contrast to previous studies with rabbits [88]. The studies in rats also used smaller ITDs, within the range a rat would be exposed during real life, unlike the experiments with rabbits. Recent experiments in rabbits support the idea that consistent BiCI stimulation can restore ITD sensitivity [98]. It should be noted that because few animals participated in the study by Rosskothen-Kuhl and colleagues, the lack of effect (i.e., no significant difference in ITD tuning between NH and late-implanted groups) in one study could have been driven by the variability in rats with NH [95].

Differences between the studies with animals and humans on the effects of early auditory deprivation could result from a lack of exposure to consistent ITDs. In humans with BiCIs, CI processors are left uncoordinated. Additionally, CI processing discards temporal fine-structure [43], which would remove the presence of fine-structure ITDs altogether, whereas this information was provided to rats in the studies by Rosskothen-Kuhl and colleagues. A recent study with the largest sample size of adult listeners with BiCIs to date (many of the same listeners that participated in the present study) showed conflicting evidence of a relationship between ITD sensitivity and age at onset of deafness when listeners were separated into groups who became deaf before or after 18 years of age [4]. This recent study makes it clear that earlier onsets of deafness are not the most robust predictor of sensitivity to ITDs, and further underscore the need for more research on this topic.

The majority of published studies imply a functionally relevant reorganization of the brainstem that occurs when the auditory system does not receive coherent bilateral stimulation during development. Results from the present study are consistent with this body of literature and demonstrate patterns that are generally consistent with previous localization experiments in adults and children with BiCIs [7, 10, 13, 14]. However, it is clear from the recently contrasting results that more work is needed to fully understand the role of early, bilateral stimulation on the ability to accurately encode spatial cues in the brainstem and midbrain. Specifically of interest is whether the binaural system can overcome the limitations of early auditory deprivation with coherent binaural stimulation later in life. The majority of listeners in the present study received BiCIs in adulthood, which makes them vastly different from new patients that are congenitally deaf or become deaf early in life and have earlier access to CIs. It is entirely possible that some of the differences observed between the earlier and later onset of deafness groups could be driven by a latent variable that has more to do with the etiology of disease that led to earlier or later deafness rather than early auditory experience.

#### Deafness during adulthood

When deafness occurs later in life, it is thought that the auditory brainstem and midbrain will have typically developed due to normal input that leads to the activity-dependent reorganization discussed in the previous section. Thus, poor localization performance should be due to other factors, or deterioration of those typically developed circuits. We therefore expected both listeners with longer periods of auditory deprivation to have poorer localization outcomes. Instead, factors related to auditory deprivation (e.g., inter-implantation delay, years of bilateral impairment) were not the most robust predictors of performance. One explanation for this weak effect is provided by Thakkar et al. [4], showing that losses in ITD sensitivity due to a longer period of bilateral deafness may be ameliorated by experience with CIs. This result also explains why experience with CIs by itself is not often associated with localization performance.

Another recent study provided evidence that deterioration of the worse ear is more important than the difference or asymmetry in function between ears. Ihlefeld et al. [99] showed that the ear with poorer temporal sensitivity could predict the amount of ITD sensitivity in adults with BiCIs. A related study in listeners with NH showed that, for comparisons of temporal information of simultaneously presented sounds within- or across-ears, poorer fidelity of temporal information in only one of the two places-of-stimulation degrades performance [100]. Together with the results of Reeder et al. [14], these studies suggest that the more important variable is not the delay between ears, but the longer length of deafness, likely leading to greater deterioration of the auditory periphery due to a lack of stimulation.

#### Aging

Another important factor that could lead to differences in performance across listeners is age at testing. Aging results in several changes to the auditory system. The loss of hair cells accelerates age-related increases in the death of auditory nerve fibers in humans [101]. Decreases in amplitude for all waves and increases in latency for early waves of the auditory brainstem response occur with increasing age in humans [102], suggesting fewer or less consistent inputs. Interestingly, the temporal resolution of individual auditory nerve fibers seems to remain intact during aging in the absence of hearing loss [103]. Thus, changes in the auditory brainstem response may be related to the loss of auditory nerve fibers associated with aging. A decrease in inhibition in the cochlear nucleus of mice also occurs with aging, resulting in poorer temporal processing [104]. The medial nucleus of the trapezoid body, which supplies inhibition to the LSO and MSO, has fewer living cells in older rats [105]. These results suggest that aging affects the inputs to the nuclei that perform binaural computations and imply that functional deficits may occur. This could explain some of the overlap in localization performance between the listeners with earlier (younger) and later (older) onsets of deafness in the present study.

The present study demonstrated some evidence of poorer localization performance for older listeners with BiCIs. However, aging was also not the most robust predictor of localization performance. Age at testing was almost perfectly confounded with years of acoustic exposure (i.e., age at second implantation). Thus, it could be that the lack of robustness in the effect of aging relates to the confound between these variables, where a longer period of acoustic exposure improves performance. This same finding was demonstrated in the same group of listeners who participated in the present study by Thakkar and colleagues when evaluating ILD sensitivity. Because the model predictions were so similar, this provides additional evidence that sound source localization in listeners with BiCIs is driven by ILDs (e.g., [33, 41]).

Older listeners with NH exhibit reduced sensitivity to ITDs [106, 107], smaller binaural masking level differences [106, 108, 109], and greater overall RMS error in localization [15] compared to younger listeners with NH. Studies measuring the perceived intracranial location of a sound based on binaural cues delivered via headphones or direct stimulation showed smaller ranges where listeners perceived a sound containing ITDs and ILDs for middle-aged [67] and older [64, 110] listeners compared to younger listeners with NH. Crucially, when large enough ITDs were provided to older listeners with NH or BiCIs in these studies, ITDs were lateralized to similar extents [64, 67]. This final result could help explain the medial localization responses to lateral targets (e.g., compressed means shape in Fig 6) observed in many listeners with BiCIs in previous studies [3, 13, 27, 40] and the present study. It may be that older listeners will respond with more lateral angles at lateral target angles if consistent, large ITDs are applied to stimuli.

### Evaluating sound source localization performance

Most studies of listeners with BiCIs evaluate the overall RMS error, generalizing across angle of presentation. However, as illustrated here (Fig 3) and discussed in several studies on sound source localization [3, 10, 13, 22, 27, 40, 42], this generalization ignores many of the aspects of listeners’ responses. Worse, it groups together listeners who perform poorly on average but show very different patterns of localization responses that are likely due to different underlying factors. Most listeners with BiCIs exhibit greater errors at far lateral angles, and as illustrated in Fig 3, the pattern of the localization function varies widely across patients. While the LSI was proposed as an alternative to average RMS error, it was highly related to average RMS error in the present study and does not provide much intuition as to the type of errors made by the listener, which could be driven by bias in responses or high levels of variability. The LSI might be more useful in tracking whether sensitivity improves for a listener with BiCIs after experience and training, for example.

A better approach for our problem was to consider the pattern of localization responses and describe where, how, and why errors occur. We demonstrated that one effective and intuitive approach to this problem was to sort localization results into different, pre-defined categories of performance. This type of analysis may be important for tracking patient progress across experience and development [23], assessing differences between monaural/unilateral and binaural/bilateral performance [3, 13], training patients to improve localization performance or remove bias [24, 25, 55], or improving CI algorithms [47, 48] and microphone arrays [49].

In addition to utility in BiCI studies, there are other populations of listeners and stimuli in which one would expect characteristic differences in localization across presentation angles. One classical example is localization of sinusoids or narrowband noise at center frequencies above or below 1500 Hz, which provides the basis for “duplex theory” [34]. With center frequencies near 1500 Hz, listeners make greater errors at far lateral angles [32, 111, 112], presumably due to the limited ability to encode interaural timing differences and small magnitude of interaural level differences.

## Limitations and conclusions

The present study had several limitations. Firstly, the stimuli used (trains of pink noise bursts) were ideal for localization, wideband, and not meaningful in temporal structure, reducing their generalizability to real-life stimuli like speech. In the real world, many different sound sources co-occur, are often band-limited, and contain temporal fluctuations, all of which may make them more difficult to localize than pink noise. Secondly, as shown in the Simulation Study portion of the S2 Appendix, 15 repetitions per target angle still resulted in some mis-classification of simulated listeners. A greater number of repetitions per target angle may have improved classification using unsupervised machine learning. It is worth considering whether testing fewer target angles with a greater number of repetitions per target angle is a better approach for future studies. Finally, and related to the second point, we do not expect that the categories from Fig 6 are the only possible categories of localization performance that exist. Instead, the categories in Fig 6 and the analysis approach outlined in the present study are intended as a first step toward a broader problem: understanding how localization responses change with different variables of interest (e.g., stimuli, patient-dependent factors, devices). While the computation time was considerable when completing simulations on a laptop, this could be improved considerably by using additional computing resources like those emerging in shared computing networks.

The results from the present experiment are consistent with the previous literature in listeners with BiCIs showing that RMS error increases at lateral target angles for all listeners. Our results specifically show mediating effect of later onset of deafness with squared target angle, where the errors were proportionally higher at medial target angles for listeners with an earlier onset of deafness. The LSI was highly related to RMS error across all target angles, and both were predicted by earlier onset of deafness, and slightly by age at testing. Parameters from a logistic function describing the difference between maximum and minimum of localization responses or slope of localization were unrelated to earlier onset of deafness and age at testing. The newly proposed method of analysis involving unsupervised machine learning revealed the novel result that listeners with an earlier onset of deafness show greater variability in their localization responses compared to those with a later onset of deafness. Together, these results suggest that earlier onset of deafness may lead to especially poor binaural encoding of sounds, resulting in less reliable responses.

## Supporting information

S1 Appendix Supplementary R code, plots, and additional analysis. This appendix contains worked examples in R, alternative analyses, plots of data not included in the paper, and considerable documentation of the code used for the unsupervised machine learning analysis.
(PDF)

S2 Appendix Machine learning methods. This appendix contains detailed mathematical descriptions of the algorithms used, as well as a simulation study detailing parameter choices, for the unsupervised machine learning analysis.
(PDF)

S1 File Regression analysis code. This code can be used in R on the S1 Dataset to reproduce the regression analyses included in this manuscript.
(R) Click here for additional data file.

S1 Dataset Regression analysis dataset: Patient-dependent factors. This is dataset can be used with S1 File to reproduce the regression analyses in this manuscript.
(CSV)

S2 Dataset Regression analysis dataset: RMS Error by target angle. This is dataset can be used with S1 File to reproduce the regression analyses in this manuscript.
(CSV)

S3 Dataset Machine learning dataset. This dataset can be used with the code stored and updated in the Github repository to reproduce the unsupervised machine learning analysis in this manuscript.
(CSV)
