# Supplementary material for: Sound source localization patterns and bilateral cochlear implants: Age at onset of deafness effects
Source: PLoS One. 2022 Feb 8;17(2):e0263516. doi: 10.1371/journal.pone.0263516 (PMC8824335; doi:10.1371/journal.pone.0263516)
Supplement: S1 Appendix — This appendix contains worked examples in R, alternative analyses, plots of data not included in the paper, and considerable documentation of the code used for the unsupervised machine learning analysis. (PDF) [file pone.0263516.s001.pdf]

Supplementary Material: R Code and Implementation

Sean R Anderson

Feb 6, 2020

S1 Introduction to Data and Approach {#S1}

S1.1 Raw and Transformed Data

This appendix contains two data sets: Raw responses from each subject in a localization experiment, denoted `raw.dat`, and localization sensitivity indices for every patient, denoted `lsi`. There are some extra variables that need to be removed from `raw.dat` for convenience.

The raw data are in the form of target angle vs. response angle. Negative and positive values correspond to leftward or rightward angles, respectively. Raw data for each individual in the study are plotted in Fig. 2 of the manuscript.

```
# Prepare R
  # Set random number table
set.seed(983)
  # Load dependencies
library(reshape2)
library(cluster)
library(ggplot2)
library(gridExtra)

# Import and Format Data
raw.dat <- read.csv('LocalizationRawData_compiled.csv', head=T)
lsi <- read.csv('LSIoutput.csv', head=T)

# Remove variables that are not of interest
drops <- c('cum_percent','Condition','early.id','later.id','simul.id')
raw.dat <- raw.dat[ , !(names(raw.dat) %in% drops)]

head(raw.dat)
```

```
##   subject Run Trial Targ.Az Resp.Az Rove.Level abs_error
## 1     IAG   4     5     -90 -80.376      1.912     9.624
## 2     IAG   5    15     -90 -76.379      0.859    13.621
## 3     IAG   5    23     -90 -75.352     -1.737    14.648
## 4     IAG   4    53     -90 -54.235      0.608    35.765
## 5     IAG   5    78     -90 -53.696     -2.319    36.304
## 6     IAG   3    64     -90 -52.998      2.379    37.002
```

As can be seen above, the data are in "long format", meaning that each column represents a different experimental trial from a particular subject. Data are next summarized by evaluating the means and standard deviations (SDs) for each subject at different target angles.

```
# Take means and SDs of response angle by subject
agg.mn <- aggregate(Resp.Az ~ subject + Targ.Az,
                 data = raw.dat,
                 FUN = mean)
agg.sd <- aggregate(Resp.Az ~ subject + Targ.Az,
```

```
                data = raw.dat,
                FUN = sd)

# Cobine mean and SD data frames
agg <- rbind(agg.mn, agg.sd)

# Code which row names indicate mean and which indicate SD
agg$mnvssd <- c(rep(1, length( agg[,1]) / 2), # mean
               rep(2, length( agg[,1]) / 2)) # SD
# Sort rows by subject
agg <- with(agg,
            agg[order(subject, Targ.Az, mnvssd), ])
```

S2 Multivariate Data Analysis: Unsupervised Learning

In this manuscript, we devised a new analysis approach to classify data from a localization experiment into categories. This was accomplished using unsupervised machine learning, comparing localization data and sorting responses from different subjects or simulations into "clusters". The goal of this section is to demonstrate results using the two potential unsupervised machine learning approaches.

Section S2.1 shows results of classification using only data from the experiment to determine groups. Section S2.2 shows results of classification using simulated data from predefined categories of performance based upon the psychophysical literature.

A total of 48 adults were tested using the sound source localization experimental paradigm described in this manuscript. The means and SDs of response angles were then treated as "features" in unsupervised learning algorithms. Data were formatted into a series of vectors (columns in the matrix) with the mean and SD of responses for each target angle, where rows correspond to each patient. A subset is shown below. Row names indicate subject codes. Column names give the target angle, an underscore, and an index to indicating whether that column contains (1) means, or (2) SDs.

```
# Convert data to wide format
dat <- acast(agg, subject ~ Targ.Az + mnvssd,
             value.var = 'Resp.Az')
# Place mean and SD columns next to each other
dat <- dat[, c(seq(1,37,2), seq(2,38,2)) ]
head(dat)
```

```
##         -90_1      -80_1      -70_1     -60_1      -50_1      -40_1
## IAG -24.45720 -38.848667 -29.688533 -30.90833 -31.440200 -36.187467
## IAJ -54.05087 -71.362533 -57.060200 -60.59520 -55.983533 -51.148933
## IAU -28.46807 -29.348533 -39.287333 -17.35920 -73.583067 -35.011867
## IAZ   2.14900   6.665667   1.666467   1.35380   3.051867   5.051933
## IBA -31.72900 -27.907667 -26.658067 -26.86193 -27.378733 -27.602400
## IBD -26.10027  -6.839533 -15.298933 -24.21507 -24.942000 -15.154867
##          -30_1      -20_1      -10_1        0_1       10_1      20_1
## IAG  20.387933  13.350133  -3.512133  17.096867  30.167467  37.27313
## IAJ -25.943933 -17.888800  -6.050933   5.756733  26.223867  29.37820
## IAU  -8.505533 -10.649733   3.335400  17.017267  38.624667  40.76453
## IAZ   2.121467   3.179067   3.408133   5.989867   2.810267   8.12860
## IBA -32.490067 -25.536333 -33.245133 -30.195133 -28.036600 -33.25687
## IBD -10.351333   4.965000  16.488867  28.190200  35.660400  39.04807
##           30_1       40_1       50_1      60_1      70_1      80_1
## IAG  33.044933  48.196533  56.356467  45.73520  35.71127  37.11360
```

```
## IAJ  48.986800  48.513000  49.933267  63.01767  64.59893  55.42187
## IAU  60.766467  52.492067  61.104733  72.55553  72.72020  71.27313
## IAZ   5.780733   5.330333   7.861133   6.71620  10.29840  14.88860
## IBA -43.498000 -35.205867 -36.204333 -40.26953 -32.88927 -32.47847
## IBD  46.115667  47.358667  50.873600  54.77433  48.68913  44.28947
##          90_1     -90_2    -80_2     -70_2     -60_2     -50_2     -40_2
## IAG  24.56147 55.730249 30.11213 41.916311 41.984907 44.398712 22.123515
## IAJ  62.82380 26.383201 12.07936 15.110314 18.555968 23.794918 23.099175
## IAU  56.83727 33.317446 24.21443 24.841192 32.000334 26.646228 25.083261
## IAZ  12.60347  3.004576 12.60420  4.037359  3.947271  2.638806  8.039249
## IBA -37.47467 13.778504 17.64215 19.656991 22.919494 17.339189 17.521188
## IBD  49.26773  7.528146 15.39586 13.333265  8.224384  8.426699  9.576480
##         -30_2     -20_2     -10_2       0_2      10_2      20_2      30_2
## IAG 53.296260 53.639929 50.186262 50.799391 50.624741 34.510645 53.508429
## IAJ 19.859355 22.001574  8.943750  7.333470 26.764091 26.664572 25.449366
## IAU 25.984258 23.584221 14.132656 23.455989 33.653009 27.955563 24.506362
## IAZ  2.505784  3.634861  3.460747  7.577437  3.299540 11.644295  9.025073
## IBA 12.920178 13.750321 15.272773 13.855971 21.424804 13.784032 16.843755
## IBD 11.468384 14.720111 11.374953  6.654543  7.371858  5.097042  8.486805
##          40_2     50_2      60_2     70_2     80_2      90_2
## IAG 37.150276 35.21338 50.736832 46.97131 46.23229 57.712629
## IAJ 20.369370 24.32082 21.117442 14.33715 19.36882 20.761634
## IAU 21.244182 25.17388 15.712888 19.67680 17.15864 22.415881
## IAZ  5.686039 10.08370  6.444186 13.28660 21.90902 14.826649
## IBA 17.185275 13.08526 16.971943 19.36793 16.48064 12.770451
## IBD  9.673378 10.16914 10.223034 10.23753  9.23530  9.385288
```

S2.1 Classification Using Experimentally Observed Data Only

Based upon the simulation study presented in the manuscript (code provided in Section S2.2), the partitioning around medoids (PAM) unsupervised machine learning algorithm was used for classification. Accordingly, Section S2.1 shows results for classification using the PAM algorithm based upon *only the data collected from subjects in the experiment.*

S2.1.1 Ideal Number of Groups

In unsupervised machine learning approaches, the number of classification groups must be determined prior to the analysis. Thus, there are two options for the experimenter; that is, the number of classification groups are determined based on: (1) Theory, or (2) Some measure of goodness of fit. The unsuperivsed machine learning algorithm will force at least one subject into each cluster. Since it is impossible to know the number of possible categories obtained from subjects in a new dataset a priori, we opt for the second approach to determine the number of classification groups; i.e., based on goodness of fit.

A recent study by Hennig and Lin (2015) proposed a method by which the number of clusters can be determined from the data using the average silhouette width, a measure of goodness-of-fit, for data classified into k clusters. The average silhouette width was compared with many simulated datasets that contained *no clustering* to determine how many clusters occur in the experimentally observed data.

We used the method from Hennig & Lin, 2015 to approximate the number of clusters within the data. The average silhouette width was calculated for different numbers of clusters with real and simulated data. Simulated data were generated in such a way that there was no difference between subjects, i.e., with no clustering to compare against clustering in experimentally observed data. Below is a plot showing the upper-bound for 99% confidence intervals based upon the simulated data. Any points from the experimentally

observed data that fall above the confidence interval provide evidence to suggest that there are at least that many clusters that occur in the dataset.

```
# Create 99% confidence intervals based on null model from Hennig & Lin, 2015

# -------------- DEFINITION OF NECESSARY FUNCTIONS ----------------
# Load dependencies
library(MASS)
library(cluster)

# Map std normal to sample distribution
Ginv <- function(x, mu, sigma){
  # Input:
  # x - vector of std normal observations
  # mu - sample mean (single number)
  # sigma - sample variance (single number)

  # Output: vector of observations from sample distribution

  # Convert z-score to proportion
  p <- pnorm(x, 0, 1)
  # Convert proportion to orig distribution
  out <- qnorm(p, mu, sigma)
  return(out)
}

# Simulate data from a model showing no clustering
# according to sampling distribution. Calculate and
# return average silhouette width for bootstap
# simulations
parametricBoot <- function(k, mu, sigma, nsim){
  # Input:
  # k - number of clusters
  # mu - vector of sample distribution means
  # sigma - vector of sample distribution variances
  # nsim - number of simulated subjects

  # Ouput: average silhouette width

  # Simulate std normal
  z <- mvrnorm(nsim,
               mu = rep(0, length(mu)),
               Sigma = cov2cor(sigma))

  sims <- matrix(data = NA,
                 nrow = nrow(z),
                 ncol = ncol(z))

  # Map null model data to sample distribution
  for (i in 1:length(z[1,])){
    sims[,i] <- Ginv(z[,i], mu[i], sqrt(sigma[i,i]))
  }

  # Complete PAM with specified number of clusters
```

```
  pm <- pam(sims, k)

  # Extract silhouette width
  asw <- pm$ silinfo $ avg.width
  return(asw)
}

# -------------- COMPARISON WITH EXPERIMENTAL DATA ----------------
# The data frame below provides a summary of the bootstrap simulations
# Results are plotted in Fig. S1.

# Specify parameters
m <- 500                # Number of simulations for confidence interval
k <- seq(2, 40)         # Vector of number of clusters
nsim <- 48              # Number of subjects in original dataset

# Get means and SDs from experimental data
mu <- apply(dat, MARGIN=2, FUN=mean)
sigma <- cov(dat)

# Initialization of output data frame
  # Other variable definitions given in loop

df <- data.frame(k = k, # Number of clusters
                 m = rep(m,length(k)),  # Number of simulated subjects
                 nsubs = rep(nsim,length(k)),
                 asw.data = vector(length = length(k)),
                 lower.boot = vector(length = length(k)),
                 mean.boot = vector(length = length(k)),
                 upper.boot = vector(length = length(k)),
                 raw.cluster = vector(length = length(k)))

for (i in 1:length(k)){
  # Simulate null model data with differing numbers of clusters
  asw.boot <- apply(matrix(k[i], nrow = m, ncol = 1),
                    MARGIN = 1,
                    FUN = parametricBoot,
                    mu = mu,
                    sigma = sigma,
                    nsim = nsim)

  # Complete PAM
  pm.dat <- pam(dat,k[i])
  # Calculate silhouette width on null data
  asw.dat <- pm.dat$ silinfo $ avg.width

  # Calculate silhouette width from raw data
  df$asw.data[i] <- asw.dat
  # Calculate lower confidence interval from simulations
  df$lower.boot[i] <- mean(asw.boot) -
    qnorm(0.995) * sd(asw.boot)
  # Calculate center confidence interval from simulations
  df$mean.boot[i] <- mean(asw.boot)
```

```
  # Calculate upper confidence interval from simulations
  df$upper.boot[i] <- mean(asw.boot) +
    qnorm(0.995) * sd(asw.boot)
  # Logical - silhouette width for PAM on experimental data
  # above confidence interval from null model
  df$raw.cluster <- asw.dat > df$upper.boot[i]
}
```

Experimentally observed data and simulated data with no clustering are compared below. 500 null data simulations were completed and analyzed with PAM. The average silhouette width was extracted from each simulated data set and used to generate a confidence interval. In Fig. S.1, the average silhouette widths for the data collected in the experiment are compared against the confidence intervals based upon the null data. The x-axis shows the number of k groups entered into PAM. The y-axis shows the average silhouette width, where a higher value indicates better goodness-of-fit.

```
# Plot silhouette width of simulations and raw data
plot(c(k,k), c(df$asw.data, df$upper.boot),
     col = c(rep('black', length(k)),
           rep('red', length(k))),
     pch = c(rep(1, length(k)),
           rep(17, length(k))),
     xlab = 'Number of Clusters',
     ylab = 'Average Silhouette Width',
     ylim = c(0,0.5),
     type = 'p',
     lwd = 2)
legend(11,0.49,
       legend = c('Experimental Data', 'Upper Bound Bootstrap'),
       col = c('black', 'red'),
       lwd = 2,
       lty = 0,
       pch = c(1, 17))
```

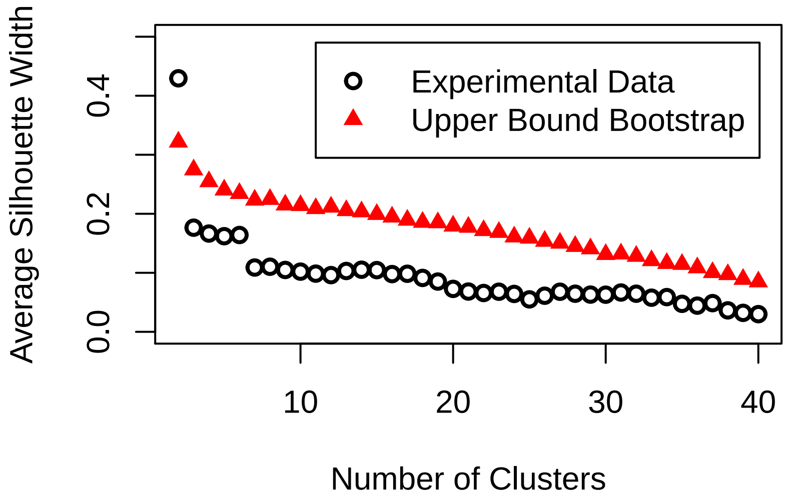

Figure S.1: Comparison of goodness-of-fit between experimental data and those with no clustering

Results from the Fig. S.1 suggest that there are 2 clusters within the dataset. Beyond two clusters, the average silhouette width is within the 99% confidence interval for classification of the simulated data containing no clustering.

S2.1.2 Visualization of Group Differences

Section S2.1.1 suggested that 2 clusters provide the best goodness-of-fit of the PAM algorithm to the experimentally observed data. The differences with respect to the pattern of localization performance for each cluster are shown below. In Fig. S.2, the y-axis represents the mean (A) or SD (B) response angle, the x-axis represents the target angle.

```
# Add mean and SD of response as separate columns
names(agg.sd) <- c('subject','Targ.Az','SD')
merged <- merge(agg.mn, agg.sd, by = c('subject','Targ.Az'))
pm <- pam(dat, 2)

# Add cluster number to data frame
clust.df <- data.frame(subject = row.names(dat),
                       cluster = pm$clustering)
merged <- merge(merged, clust.df, by = 'subject')
```

```
# Visualize means
ggplot(merged, aes(Targ.Az, Resp.Az)) +
  geom_point() +
  aes(colour = subject) +
  facet_wrap( ~as.factor( pm$clustering)) +
  xlab('Target Angle') +
  ylab('Mean Response Angle') +
  theme_bw()

# Visualize SDs
ggplot(merged, aes(Targ.Az, SD)) +
  geom_point() +
  aes(colour = subject) +
  facet_wrap( ~as.factor( pm$clustering)) +
  xlab('Target Angle') +
  ylab('SD of Response') +
  theme_bw()
```

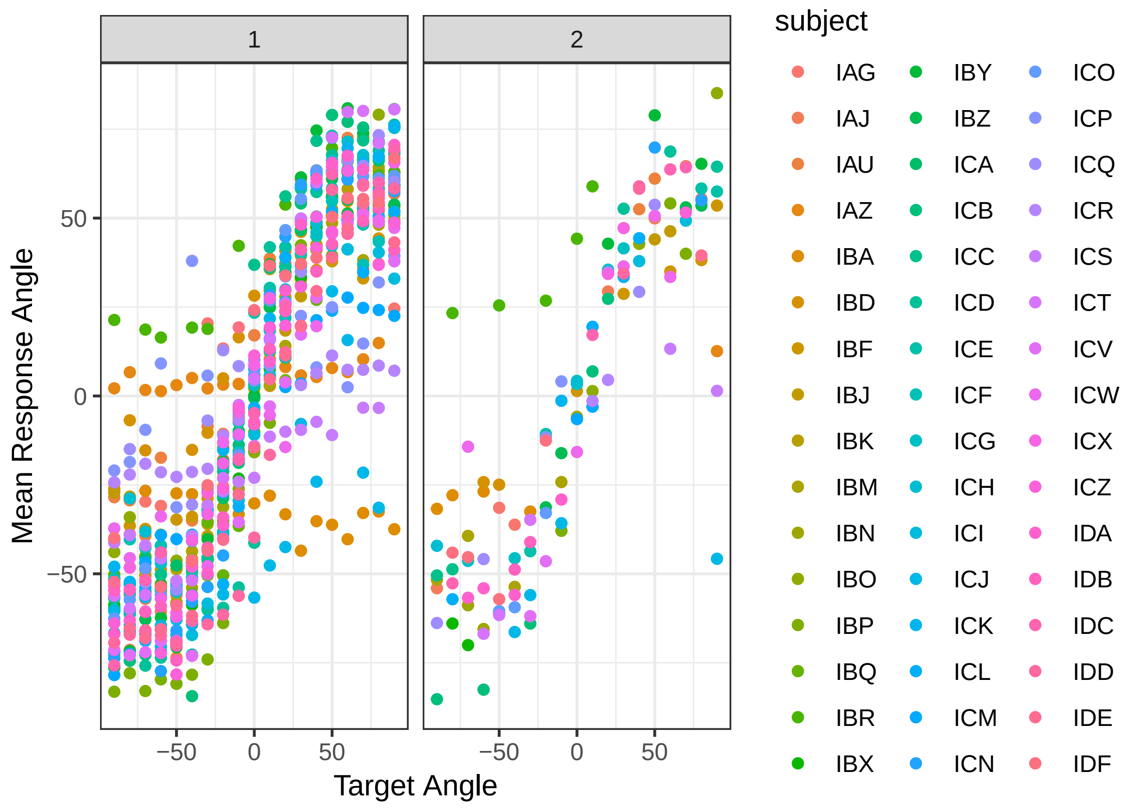

(a) Response means for clusters 1 and 2

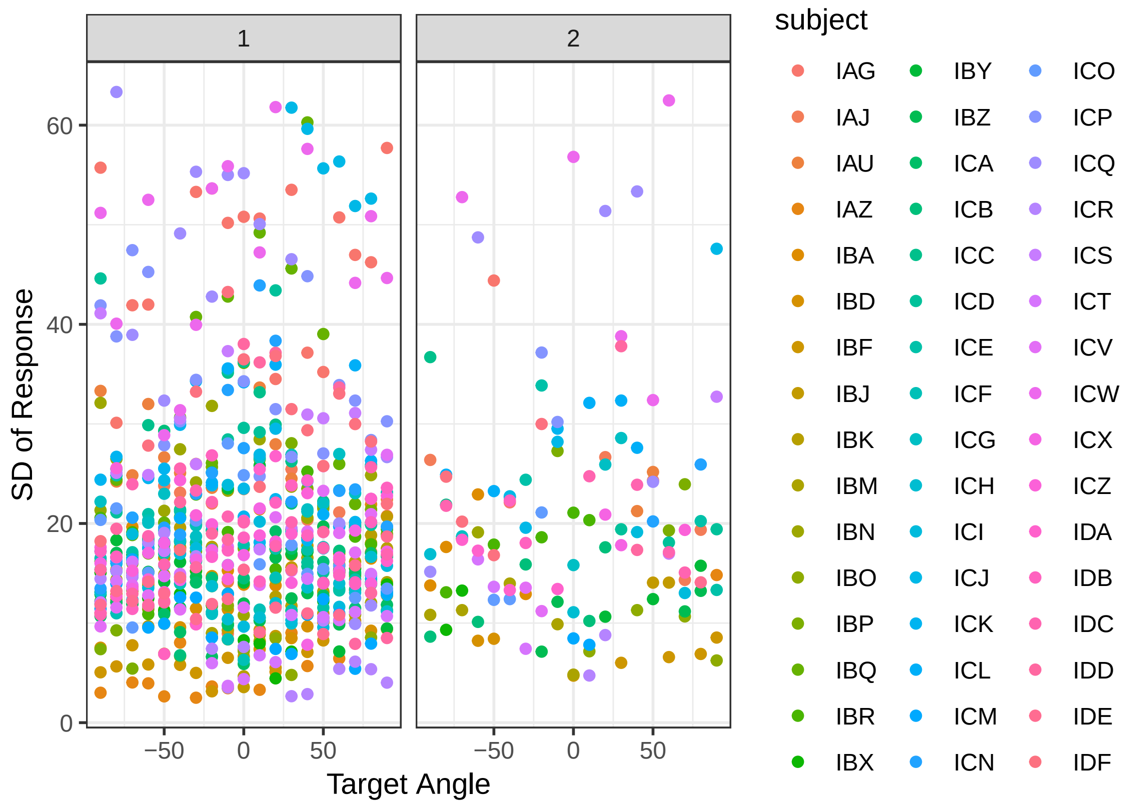

(b) Response SDs for clusters 1 and 2

Figure S.2: Visualization of group differences; group numbers shown in top of panels

From the plots above, no clear trends stand out with respect to different clusters from the experimental dataset. It is not immediately clear what aspects of the response are being used to classify patients into each cluster. Thus, these results imply that the analysis approach in Section S2.1 falls short of the goal: to classify experimentally observed data into meaningfully different categories.

S2.2 Revised Approach: Simulated Data Based on Apriori Categories

The method examined in Section S2.1, while having the strength of being data-driven, failed to demonstrate meaningful results. Recent reports (Reiss et al., 2016; Grieco-Calub and Litovsky, 2010) in our field have attempted using statistical classification criteria to distinguish between the shapes of functions from different populations of patients. However, as noted in our manuscript, these approaches have the same limitation as Section S2.1, i.e., they are limited by the data observed in the experiment.

An alterantive approach is to define groups that have been demonstrated in the literature, simulate those groups, and to determine into which group each person in the experimental dataset falls. The strength of the methods employed by Reiss et al., 2016 and Grieco-Calub and Litovsky, 2010 is comparison of data based upon important features. Different types of localization performance are discussed by Zheng et al. (2015). Thus, we attempted to form groups based on their discussion. For additional information, see the discussion section of our manuscript to which this supplement accompanies. Below is an illustration of these groups:

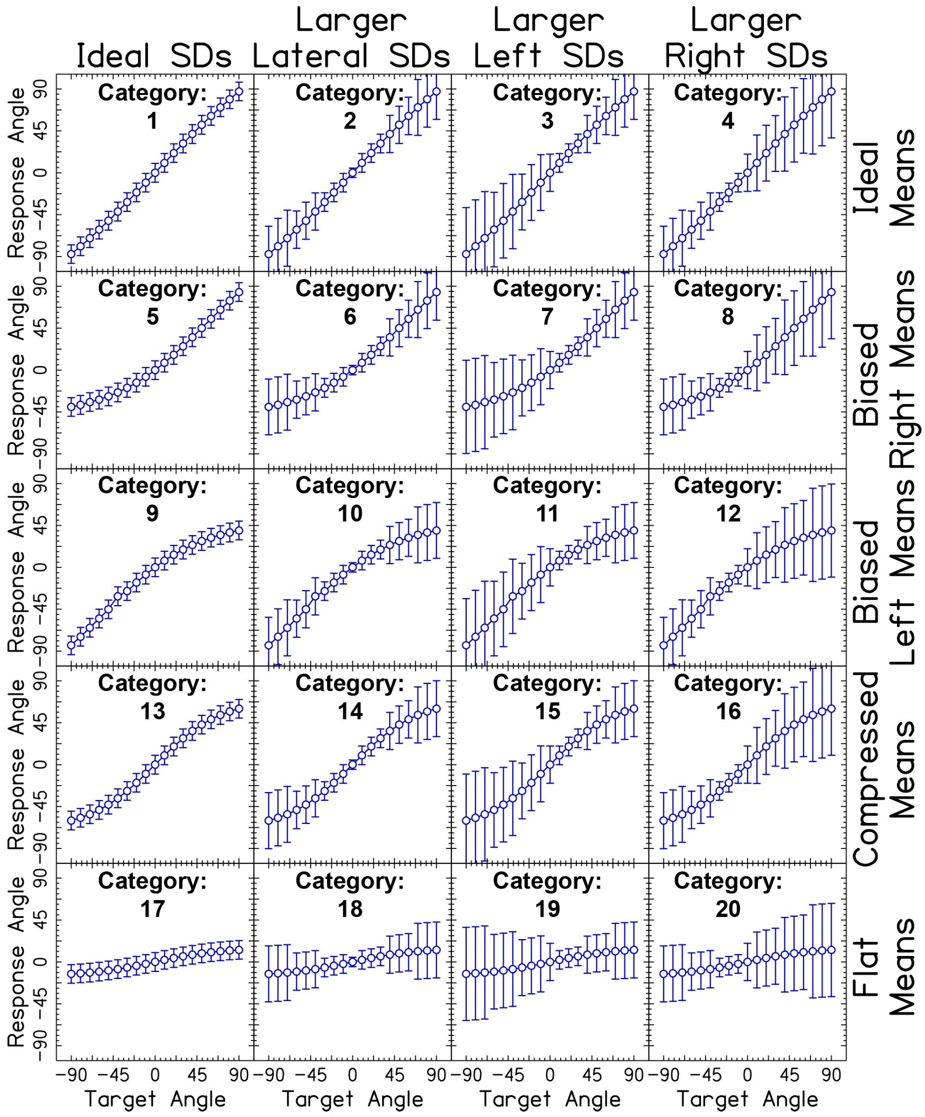

Figure S.3: Categories of localization responses; used as basis for simulations

In order for simulations of each of the above categories to be complete, a series of useful helper functions must be defined:

```
# Second lapply function
  # Allows for lapply over lapply
  # Built for and tested with data frames
dubApply <- function(dat,fu, ...){
  # Input:
  # dat - list
  # fu - function

  # Output: data object with function fu applied over list within list

  FUN <- match.fun(fu)
  if (!is.vector(dat) || is.object(dat))
```

```
    dat <- al.list(dat)
  .Internal( lapply( dat, fu))
}

# Take mean and SD of each column
colMeanSd <- function(df){
  # Input: df - data frame

  # Output: data frame of means and SDs from each column of input data frame

  # Calculate means and SDs from each column
  m <- apply(df, MARGIN = 2, FUN = mean)
  s <- apply(df, MARGIN = 2, FUN = sd)

  # Concatenate columns
  out <- as.data.frame( c(m,s))
  return(out)
}

# Bind all columns of data frames in a list
combListDf <- function(l){
  # Input: l - list of data frames

  # Output: large data frame concatenated from list

  out <- do.call('cbind', l)
  return(out)
}

# Returns matrix that can be input to clustering algorithms
clusFormat <- function(dat){
  # Input: dat - raw responses from participant/simulation

  # Output: matrix where rows are observations/subjects,
  # cols are features

  # Take means and SDs of each column
  l <- lapply(dat, FUN = dubApply, fu=colMeanSd)

  # Combine means and SDs from each sample
  l <- lapply(l, FUN = combListDf)

  # Transpose matrices
  out <- lapply(l, FUN = t)
  return(out)
}
```

Using the helper functions defined above, two functions are now defined: (1) `simLoc` generically simulates data according to response means and standard deviations specified by the user, and (2) `simLocData` defines the means and standard deviations from Fig. S.3 and calls `simLoc` to generate data from each category.

```
# Simulate localization data according to categories defined
# by user.
simLoc <- function(angle, rmeans, rsds, n, lsiz){
```

```r
  library(truncnorm)
  # Input:
  # angle - vector of target angles
  # rmeans - vector of response means at each target angle
  # rsds - vector of response SDs at each target angle
  # n - number of responses per target speaker
  # lsiz - number of simulated subjects per category
    # also indicates size as output list

  # Output: data frame of means and SDs at each target angle

  # Generate data from truncated normal distribution
  x <- as.data.frame( mapply( rtruncnorm,
              mean = rmeans,
              sd = rsds,
              n = n*lsiz,
              a = -90, b = 90))

  # Name each column according to mean
  names(x) <- as.character(angle)

  # Split data into lists
  f <- sort( rep( seq(1,lsiz), n))
  l <- split(x,f)
  return(l)
}
```

Here we provide an illustration of how `simLoc` works. Suppose it is the goal of the experimenter to simulate data where response means align well with target speakers and SDs are uniform and small (as in normal-hearing). To use `simLoc`, we first define the *population distribution* from which simulated subjects will be drawn. In this example, we will use the `SSfpl` function, a four-parameter logistic function, to simulate these data. The parameters in `SSfpl` are set such that they result in near-perfect performance.

First, the target speaker locations are specified. Then, population means are determined using the `SSfpl` function based on target speakers. The SD at each target speaker location is defined along number of repetitions per target speaker and number of simulated subjects outright in this example, but could be determined by some function in future applications. Finally, `n` data points are sampled from the population distribution for each target speaker location using a truncated normal distribution (`truncnorm`), where responses are limited to ±90 degrees. The results of `lsiz = 20` simulated subjects are plotted in Fig. S.4.

```r
# Sequence of target speaker locations
x <- seq(-90, 90, 10)
# Sequence of means equal to target speakers
y <- SSfpl(x, -180, 180, 0, 85)
# Sequence of SDs equal across target speakers
SDs <- rep(10, length(x))
# Number of repetitions per target speaker
n <- 15
# Number of subjects simulated
lsiz <- 20

# Simulate data
samp.dat <- lapply(n, FUN = simLoc, angle = x,
    rmeans = y, rsds = rev(SDs), lsiz = lsiz)
samp.clus <- clusFormat(samp.dat)
```

```r
# Jitter target speaker values to show differences between simulated subjects
x <- jitter( sort( rep( x, nrow(samp.clus[[1]]))))
plot(x = x,
# Plot response means
    y = samp.clus[[1]][,1:19],
    xlim = c(-90, 90),
    ylim = c(-90, 90),
    xlab = 'Target Angle',
    ylab = 'Response Angle')
# Add error bars to response means
arrows(x,c(samp.clus[[1]][,1:19] + samp.clus[[1]][,20:38]),
       x,c(samp.clus[[1]][,1:19] - samp.clus[[1]][,20:38]),
       code = 0)
# Show perfect performance reference line
abline(a = 0, b = 1, col = 'red')
legend(-95,95,
       legend = c('Simulated Data','Perfect Results'),
       col = c('black','red'),
       lwd = 2,
       lty = 1,
       pch = -1)
```

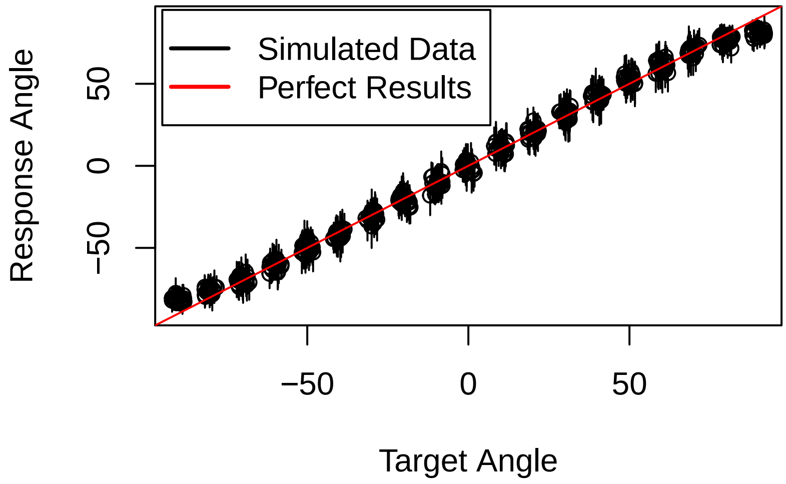

Figure S.4: Example of simulated data; red line shows perfect performance

Fig. S.4 shows data from 20 simulated subjects using the `simLoc` function. The x-axis represents the target angle, and y-axis represents the response angle, where points indicate the mean and error bars represent ±1 SD. From Fig. S.4 it can be seen that there are small differences between each simulated subject. These differences will be even more apparent when SDs are larger or the number of repetitions per target speaker are smaller. Thus, one strength of the current approach is that it uses bootstrap simulations and accounts for the differences between subjects that can be due to sampling error.

Using `simLoc`, the function `simLocData` simulated data using categories of localization responses defined in Fig. S.3. `simLocData` can be updated by future experimenters to edit, add, or remove categories that they deem appropriate. Below, `simLocData` is defined. In Section S2.2.1 it is then used to complete a simulation study and examine the accuracy of classification using simulated data.

```r
# Simulated classes are defined and simulations are completed for
# each category.
```

```r
simLocData <- function(n, lsiz){
  # Input:
  # n - number of repetitions per target speaker
  # lsiz - number of simulated subjects per category
    # also indicates length of list

  # Ouput: data frame of means and SDs from each simulated category

  # Target speaker locations
  x <- seq(-90, 90, 10)
  # Sequences of SDs for ideal and larger lateral categories
  SDs.eq <- rep(10, length(x))
  SDs.un <- c(30, 30, 30, 20, 20, 20, 10, 10, 10,
              5,  10, 10, 10, 20, 20, 20, 30, 30, 30)
  SDs.al <- c(50, 50, 50, 40, 40, 40, 30, 30, 30,
              20, 10, 10, 10, 20, 20, 20, 30, 30, 30)

# ------------ Generate Data ------------
  # Each sub-section is separated by patterns of means.
  # New cases can be added or adjusted here by the user.

  # Ideal means
  y <- SSfpl(x, -180, 180, 0, 85)
  dat.ideal.eq <- lapply(n, FUN = simLoc, angle = x,
            rmeans = y, rsds = SDs.eq, lsiz = lsiz)
  ideal.eq <- clusFormat(dat.ideal.eq)
  dat.ideal.un <- lapply(n, FUN = simLoc, angle = x,
            rmeans = y, rsds = SDs.un, lsiz = lsiz)
  ideal.un <- clusFormat(dat.ideal.un)
  dat.ideal.al <- lapply(n, FUN = simLoc, angle = x,
            rmeans = y, rsds = SDs.al, lsiz = lsiz)
  ideal.al <- clusFormat(dat.ideal.al)
  dat.ideal.ar <- lapply(n, FUN = simLoc, angle = x,
            rmeans = y, rsds = rev(SDs.al), lsiz = lsiz)
  ideal.ar <- clusFormat(dat.ideal.ar)

  # Biased left means
  y <- SSfpl(x, -50, 150, 55, 50)
  dat.asyml.eq <- lapply(n, FUN = simLoc, angle = x,
            rmeans = y, rsds = SDs.eq, lsiz = lsiz)
  asyml.eq <- clusFormat(dat.asyml.eq)
  dat.asyml.un <- lapply(n, FUN = simLoc, angle = x,
            rmeans = y, rsds = SDs.un, lsiz = lsiz)
  asyml.un <- clusFormat(dat.asyml.un)
  dat.asyml.al <- lapply(n, FUN = simLoc, angle = x,
            rmeans = y, rsds = SDs.al, lsiz = lsiz)
  asyml.al <- clusFormat(dat.asyml.al)
  dat.asyml.ar <- lapply(n, FUN = simLoc, angle = x,
            rmeans = y, rsds = rev(SDs.al), lsiz = lsiz)
  asyml.ar <- clusFormat(dat.asyml.ar)

  # Biased right means
  y <- SSfpl(x, -150, 50, -55, 50)
```

```
  dat.asymr.eq <- lapply(n, FUN = simLoc, angle = x,
            rmeans = y, rsds = SDs.eq, lsiz = lsiz)
  asymr.eq <- clusFormat(dat.asymr.eq)
  dat.asymr.un <- lapply(n, FUN = simLoc, angle = x,
            rmeans = y, rsds = SDs.un, lsiz = lsiz)
  asymr.un <- clusFormat(dat.asymr.un)
  dat.asymr.al <- lapply(n, FUN = simLoc, angle = x,
            rmeans = y, rsds = SDs.al, lsiz = lsiz)
  asymr.al <- clusFormat(dat.asymr.al)
  dat.asymr.ar <- lapply(n, FUN = simLoc, angle = x,
            rmeans = y, rsds = rev(SDs.al), lsiz = lsiz)
  asymr.ar <- clusFormat(dat.asymr.ar)

  # Compressed means
  y <- SSfpl(x, -70, 70, 0, 35)
  dat.compr.eq <- lapply(n, FUN = simLoc, angle = x,
            rmeans = y, rsds = SDs.eq, lsiz = lsiz)
  compr.eq <- clusFormat(dat.compr.eq)
  dat.compr.un <- lapply(n, FUN = simLoc, angle = x,
            rmeans = y, rsds = SDs.un, lsiz = lsiz)
  compr.un <- clusFormat(dat.compr.un)
  dat.compr.al <- lapply(n, FUN = simLoc, angle = x,
            rmeans = y, rsds = SDs.al, lsiz = lsiz)
  compr.al <- clusFormat(dat.compr.al)
  dat.compr.ar <- lapply(n, FUN = simLoc, angle = x,
            rmeans = y, rsds = rev(SDs.al), lsiz = lsiz)
  compr.ar <- clusFormat(dat.compr.ar)

  # Flat means
  y <- SSfpl(x, -15, 15, 0, 35)
  dat.flatt.eq <- lapply(n, FUN = simLoc, angle = x,
            rmeans = y, rsds = SDs.eq, lsiz = lsiz)
  flatt.eq <- clusFormat(dat.flatt.eq)
  dat.flatt.un <- lapply(n, FUN = simLoc, angle = x,
            rmeans = y, rsds = SDs.un, lsiz = lsiz)
  flatt.un <- clusFormat(dat.flatt.un)
  dat.flatt.al <- lapply(n, FUN = simLoc, angle = x,
            rmeans = y, rsds = SDs.al, lsiz = lsiz)
  flatt.al <- clusFormat(dat.flatt.al)
  dat.flatt.ar <- lapply(n, FUN = simLoc, angle = x,
            rmeans = y, rsds = rev(SDs.al), lsiz = lsiz)
  flatt.ar <- clusFormat(dat.flatt.ar)

# ------------ Reformat Data ------------
  # Takes data frames from each simulated class
  # and combines into one larger data frame.
  # New classes also need to be added here if
  # they are introduced by the user.

  out <- do.call('rbind',list(
    ideal.eq[[1]], ideal.un[[1]], ideal.al[[1]], ideal.ar[[1]],
    asyml.eq[[1]], asyml.un[[1]], asyml.al[[1]], asyml.ar[[1]],
    asymr.eq[[1]], asymr.un[[1]], asymr.al[[1]], asymr.ar[[1]],
```

```
    compr.eq[[1]], compr.un[[1]], compr.al[[1]], compr.ar[[1]],
    flatt.eq[[1]], flatt.un[[1]], flatt.al[[1]], flatt.ar[[1]]))
  return(out)
}
```

S2.2.1 Simulation Study

Before applying these methods to experimental data, we first wanted to evaluate performance of different clustering algorithms where the correct clustering is known a priori. Since data are simulated from the functions shown in each panel of Fig. S.3, there is a 'correct' category to which each simulated subject belongs. Several *external evaluation* criteria have been defined to evaluate unsupervised learning algorithms when there are a priori groups in the data (Manning et al., 2009). These criteria can be used to measure the effectiveness of unsupervised machine learning algorithms by comparing clustering performance against chance performance. A description and corresponding equations are given in the Methods section of our manuscript.

Because different sample sizes are collected in different experiments and laboratories, it was important to evaluate how classification accuracy to the categories in Fig. S.3 is affected by sampling error. Tables of each of these external evaluation criteria with differing numbers of observations per presentation angle (rows) and number of simulated subjects (columns) are given below.

```
# Calculate purity of cluster
purity <- function(y, yprime){
  # Input:
  # y - true clustering
  # yprime - algorithmic clustering

  # Output: purity bounded [0,1]

  # Load required library
  library(caret)

  # Generate confusion matrix
  cm <- confusionMatrix( as.factor( yprime),
                         as.factor( y ))

  # Find mode of each cluster
  maxs <- apply(cm$table, MARGIN = 2, FUN = max)

  # Compute cluster similarity statistics
  out <- sum(maxs / (length(y)))
  return(out)
}

# Computes different external evaluation metrics.
# Presents results in a summary table.
externalEval <- function(
  clus.mat, k, gindex, cmtype = 2, print.out = F, alg = 'PAM'){
  # Input:
  # clus.mat - clustering matrix
    # rows are subjects
    # columns are features (means and SDs)
  # k - number of clusters
```

```r
# gindex - true clustering vector
# cmtype - type of confusion matrix to be used
# print.out - logical - whether to print summary table
# alg - clustering algorithm to be used
  # current options are 'PAM' or 'kmeans'

# Output: data frame of external evaluation criteria

# Load dependencies
library(caret)
library(aricode)

# Perform clustering
  # Other unsupervised machine learning algorithms
  # could be implemented here by user
if (alg == 'PAM'){ # Default: PAM
  pm <- pam(clus.mat, k)
  # vector indicating to which cluster each subject belongs
  clustering <- pm$clustering
} else if (alg == 'kmeans'){ # Alternative: k-means
  km <- kmeans(clus.mat, k)
  # vector indicating to which cluster each subject belongs
  clustering <- km$cluster
}

# Get confusion matrix object
cm <- confusionMatrix( as.factor( clustering),
                       as.factor( gindex))

# Compute cluster similarity statistics
pure <- purity(gindex, clustering)
nmi <- NMI(gindex, clustering, variant = 'sqrt')
randi <- RI(gindex, clustering)

if (print.out){
  cat( paste0( 'Clustering purity:\n',
               pure, '\n\n'))
  cat( paste0( 'Normalized mutual information:\n',
               nmi, '\n\n'))
  cat( paste0( 'Rand Index:\n',
               randi, '\n\n'))
}

# Print confusion matrix
if (cmtype == 1 && print.out == T){ # Preserve rows
  row.index <- apply(cm$table, MARGIN = 2, FUN = which.max)
  print(cm$table[ ,as.numeric( names( sort( row.index)))])
} else if (cmtype == 2 && print.out == T){ # Preserve columns
  col.index <- apply(cm$table, MARGIN = 1, FUN = which.max)
  print(cm$table[as.numeric( names( sort( col.index))),])
}

# Create data frame of external evaluation criteria
```

```
    # stringsAsFactors must be set to FALSE to prevent coercing
    # alg to a numeric format
  out <- data.frame(purity = pure,
                    nmi = nmi,
                    rand.index = randi,
                    alg = alg,
                    stringsAsFactors = F)
  return(out)
}
```

Using the external evaluation functions above and `simLocData`, clustering performance was evaluated for simulated data, where perfect clustering would result in all data simulated from the same category being sorted into the same cluster, resulting in a value of 1 for each criterion. To examine clustering performance, the number of simulated subjects and repetitions per target speaker were adjusted. For additional details, see the Simulation Study portion of the methods in the manuscript that this supplement accompanies.

To complete the simulation study, data were simulated iteratively using `simLocData` 2000 times with the criteria indicated in the top section below. A summary table showing the values of different external evaluation criteria appear after the next code chunk. Note that due to the large number of simulations and implementation of the simulations within nested `for` loops, generating the data takes several hours. The time that it takes for each portion below is reported beneath each code chunk.

```
now <- Sys.time()

# Simulation specifications
nsims <- 2000                      # Number of simulation iterations
n.loc.conds <- 20                  # Number of possible categories
n <- c(5, 10, 15, 20, 50, 100)  # Repetitions per target angle
nsubs <- c(5, 10, 20, 50, 100)  # Number of simulated subjects

# Initialize data frames for output
v <- vector(length = (length(n) * length(nsubs)))
sum.df <- data.frame(n = v,
                     nsubs = v,
                     purity = v,
                     nmi = v,
                     rand.index = v,
                     alg = v)
km.df <- data.frame(n = v,
                    nsubs = v,
                    purity = v,
                    nmi = v,
                    rand.index = v,
                    alg = v)

# Initialize loop index
m <- 0
for (i in 1:nsims){
  for (j in 1:length(n)){
    for (k in 1:length(nsubs)){
      # Increase loop index
      m <- m + 1

      # Simulate data from all possible categories
      clus.mat <- simLocData(n[j], nsubs[k])
```

```r
      # Create vector of group numbers based on shuffling
      gindex <- sort( rep( 1 : n.loc.conds, nrow(clus.mat) / n.loc.conds))

      # Randomly shuffle rows in data frame
      shuffi <- sample( nrow( clus.mat))
      clus.mat <- clus.mat[shuffi,]
      # Update group numbers
      gindex <- gindex[shuffi]

      # Add new data to summary data frame
      sum.df[m,1] <- n[j]
      sum.df[m,2] <- nsubs[k]
      sum.df[m,3:6] <- externalEval(clus.mat,
              n.loc.conds,
              gindex,
              cmtype=2,
              alg='PAM')

      # Add new data to comparison data frame using alternative, k-means algorithm
      km.df[m,1] <- n[j]
      km.df[m,2] <- nsubs[k]
      km.df[m,3:6] <- externalEval(clus.mat,
              n.loc.conds,
              gindex,
              cmtype=2,
              alg='kmeans')
    }
  }
}

# Take mean over simulations
sum.mean <- aggregate(. ~ n + nsubs + alg, FUN = mean, data = sum.df)
# Rename columns to reflect mean
names(sum.mean) <- c('n','nsubs','alg','purity.mean','nmi.mean','rand.index.mean')
# Take SD over simulations
sum.sd <- aggregate(. ~ n + nsubs + alg, FUN = sd, data = sum.df)
# Rename columns to reflect mean
names(sum.sd) <- c('n','nsubs','alg','purity.sd','nmi.sd','rand.index.sd')

# Same steps for alternative k-means algorithm
km.mean <- aggregate(. ~ n + nsubs + alg, FUN = mean, data = km.df)
names(km.mean) <- c('n','nsubs','alg','purity.mean','nmi.mean','rand.index.mean')
km.sd <- aggregate(. ~ n + nsubs + alg, FUN = sd, data = km.df)
names(km.sd) <- c('n','nsubs','alg','purity.sd','nmi.sd','rand.index.sd')

# Assemble mean and SD in same data frame
sum.out <- merge(sum.mean,sum.sd,by=c('n','nsubs','alg'))
km.out <- merge(km.mean,km.sd,by=c('n','nsubs','alg'))
out <- rbind(sum.out,km.out)

# Write out summary CSV file
write.csv(out, file = 'simulationStudyCIs.csv',
          col.names = T, row.names = F)
```

```r
# Print runtime for code chunk
difftime(Sys.time(),now)
```

```
## Time difference of 1.959704 days
```

Now that simulations are complete, we define some convenient summary functions that print a table of the results for the experimenter. These data are summarized in Fig. 8 of the manuscript.

```r
# Print summary of external evaluation statistics
sumTables <- function(x){
  # Input:
  # x - matrix with columns corresponding to number of
  # repetitions per target speaker, number of simulated
  # subjects, purity, normalized mutual information,
  # rand index, and clustering algorithm. Rows correspond
  # to different numbers of target speakers. Columns
  # correspond to different numbers of subjects.

  helper <- function(f, n, nsubs){
    # Helper sub-function; formats text for output
    mat <- matrix(f,
         nrow = length(n),
         ncol = length(nsubs),
         byrow = F)
    row.names(mat) <- paste0('n=', n)
    colnames(mat) <- paste0('#subs=', nsubs)
    print(mat)
  }

  cat(paste0('Completed using ', x$alg[1], ' algorithm.\n'))
  n <- unique(x$n)
  nsubs <- unique(x$nsubs)
  cat('Purity:\n')
  helper(x$purity, n, nsubs)
  cat('\nNMI:\n')
  helper(x$nmi, n, nsubs)
  cat('\nRand Index:\n')
  helper(x$rand.index, n, nsubs)
}

# Print summaries for PAM and k-means
sumTables(sum.mean)
```

```
## Completed using PAM algorithm.
## Purity:
##        #subs=5  #subs=10  #subs=20  #subs=50 #subs=100
## n=5   0.749045 0.7162000 0.7095562 0.7140135 0.7232545
## n=10  0.896480 0.8987300 0.9051313 0.9135865 0.9196778
## n=15  0.951875 0.9545550 0.9596038 0.9653080 0.9686155
## n=20  0.975710 0.9784625 0.9819625 0.9845435 0.9864975
## n=50  0.999335 0.9995800 0.9997050 0.9998125 0.9998463
## n=100 1.000000 1.0000000 0.9999988 1.0000000 1.0000000
##
## NMI:
##         #subs=5  #subs=10  #subs=20  #subs=50 #subs=100
```

```
## n=5   0.7881349 0.7667279 0.7541586 0.7488250 0.7518196
## n=10  0.9170543 0.9148492 0.9137095 0.9151502 0.9181408
## n=15  0.9617070 0.9603021 0.9607284 0.9621140 0.9636232
## n=20  0.9805566 0.9800627 0.9807569 0.9811731 0.9822547
## n=50  0.9994221 0.9995419 0.9996146 0.9997001 0.9997191
## n=100 1.0000000 1.0000000 0.9999983 1.0000000 1.0000000
##
## Rand Index:
##         #subs=5  #subs=10  #subs=20  #subs=50 #subs=100
## n=5   0.9489883 0.9536198 0.9558000 0.9577461 0.9591738
## n=10  0.9806907 0.9830046 0.9843043 0.9857429 0.9867273
## n=15  0.9910224 0.9920822 0.9929467 0.9938295 0.9943494
## n=20  0.9955354 0.9961478 0.9967050 0.9971140 0.9974476
## n=50  0.9998791 0.9999201 0.9999426 0.9999629 0.9999694
## n=100 1.0000000 1.0000000 0.9999998 1.0000000 1.0000000
```

```r
sumTables(km.mean)
```

```
## Completed using kmeans algorithm.
## Purity:
##        #subs=5  #subs=10  #subs=20  #subs=50 #subs=100
## n=5   0.796415 0.8062725 0.8242038 0.8370240 0.8447135
## n=10  0.895440 0.8994425 0.9036900 0.9077705 0.9079632
## n=15  0.925030 0.9244600 0.9241712 0.9210680 0.9196853
## n=20  0.936000 0.9326350 0.9294475 0.9256280 0.9220325
## n=50  0.943815 0.9374125 0.9327287 0.9266620 0.9234673
## n=100 0.942610 0.9353650 0.9287113 0.9233300 0.9197835
##
## NMI:
##         #subs=5  #subs=10  #subs=20  #subs=50 #subs=100
## n=5   0.8363897 0.8333796 0.8404348 0.8465485 0.8502293
## n=10  0.9080442 0.9069214 0.9068733 0.9087558 0.9096695
## n=15  0.9276498 0.9248395 0.9235694 0.9229128 0.9231220
## n=20  0.9347150 0.9307441 0.9280963 0.9268707 0.9256672
## n=50  0.9369757 0.9315653 0.9285320 0.9262970 0.9260136
## n=100 0.9344143 0.9283934 0.9232608 0.9218830 0.9217776
##
## Rand Index:
##         #subs=5  #subs=10  #subs=20  #subs=50 #subs=100
## n=5   0.9599937 0.9624608 0.9653676 0.9679387 0.9692784
## n=10  0.9728338 0.9733500 0.9732957 0.9740951 0.9746272
## n=15  0.9759087 0.9751610 0.9746830 0.9747621 0.9748852
## n=20  0.9767041 0.9752296 0.9744148 0.9741399 0.9737307
## n=50  0.9753287 0.9730052 0.9717832 0.9709588 0.9708635
## n=100 0.9738820 0.9714212 0.9691510 0.9686137 0.9687429
```

Rows in the tables above indicate the number of repetitions per target speaker. Columns represent the number of simulated subjects. From the tables above, it can be seen that PAM results in more accurate clustering of data simulated from the categories in Fig. S.3 than k-means in most cases.

S2.2.2 Evaluation Using Experimental Data

Results from the simulation study in Section S.2.1 suggest that consistency of the PAM clustering algorithm reaches optimal classification performance near 20 observations per presentation angle and is similar depending

upon the number of simulated subjects. This new analysis will sort data into the most similar categories, and therefore chance error due to samping must be accounted for. Thus, the best approach would be to use a very high number of comparisons, repeat the analysis many times, and take the mode.

Below the observed data are combined with the simulated data to determine the cluster for which the observed data is most similar one sample at a time. We chose to provide 50 examples of each category and repeat the process 50 times for each subject.

Below we report the category number (which can be determined from left to right, then top to bottom in the simulated data above), the proportion of the time that subject was assigned to that category, and a criterion for the inter-rater reliability across subjects and repetitions. Inter-rater reliability, or consistency of the categories assigned to each subject, was quantified using Krippendorf's alpha. The value is printed and bounded between 0 and 1, where 1 indicated consistent category assignment for every subject in the dataset across the 50 iterations completed. The run time is printed below this code chunk.

```
now <- Sys.time()

# Number of simulated datasets compared against each
# subject in experimental data
nrep <- 30

# Assigns categories from Fig. S.2, defined in simLocData,
# to subjects based on their response means and SDs at
# each target angle. Category assignment is based on the
# mode of simulated subjects' categories that are
# classified into the same cluster. Process is completed
# iteratively for each subject and repeated nrep times
# with newly simulated data.
classAssignment <- function(dat, nrep = 30, n.sim.cases){
# Input:
# dat - clutering data frame
  # rows are subjects
  # columns are 'features'
  # i.e., mean/SD response angles for each target angle
# nrep - number of iterations for which classification
  # is performed for each subject.
  # Each iteration is completed using newly simulated data
    # from each category.
# n.sim.cases - number of simulated subjects per category
  # based on categories in Fig. S2

# Output: list containing the following elements:
# class.dist - data frame containing:
  # mode of clustering for each subject
  # proportion of time that mode occurred
    # prop = # mode / nrep
# class.df - list whose length = nrep
  # made of data frames
  # each row corresponds to a different subject
  # Variable 'class' in each data frame indicates
    # the category to which that subject was assigned
    # during the jth iteration.

  # Initialize list
  class.df <- list(length = nrep)
```

```r
for (j in 1:nrep){
  # Initialize df in jth element of list
  class.df[[j]] <- data.frame(class = vector(length = nrow(dat)),
                              purity = vector(length = nrow(dat)),
                              n = vector(length = nrow(dat)))
  # Simulate data from all possible categories
    # First argument = repetitions per target speaker
  sim.dat <- simLocData(15, n.sim.cases)

  for (i in 1:nrow(dat)){
    # Bind data frames from list into large data frame
    clus.mat <- rbind(sim.dat, dat[i,])
    # Create vector of group numbers based on shuffling
    gindex <- c( sort( rep( 1:20, nrow(sim.dat) / 20)), NA)
    # Randomly shuffle rows in data frame
    shuffi <- sample( nrow( clus.mat))
    clus.mat <- clus.mat[shuffi,]
    # Update group numbers
    gindex <- gindex[shuffi]
    # Find case where gindex == NA
      # This indicates data from experiment
    item.index <- match(NA, gindex)

    # Perform PAM
      # k = 20 since there are 20 simulated categories
    pm <- pam(clus.mat,20)
    # Store the category from each observation
      # Takes the mode of the cluster to which
        # the ith subject was assigned.
      # 'table' calculates number of elements with
        # a particular value.
      # Values input to table = category to which
        # simulated data in same cluster are assigned.
      # Ordered from greatest to least
      # First element reflects greatest number of obs
    class.df[[j]]$class[i] <- as.numeric( names( sort(
    # Only data from experiment
      # item.index is logical indicating observation was
        # from experiment.
      table( gindex[pm$clustering == pm$clustering[item.index]]),
      decreasing = T))[1])

    # Find the size of each cluster
    class.df[[j]]$n[i] <- length( gindex[
      pm$clustering == pm$clustering[item.index]])
    # Compute the purity based on simulated data
    class.df[[j]]$purity[i] <- max( table( gindex[
      pm$clustering == pm$clustering[item.index]])) / (class.df[[j]]$n[i] - 1)
  }
}

# Initialize data frame
class.dist <- data.frame(class = vector(length = nrow(dat)),
```

```
                              prop = vector(length = nrow(dat)))

  # Extract category assignments for each subject during each iteration in nrep
  mat <- matrix( unlist( lapply( class.df,
            FUN = function(x){ x$class })), nrow = nrow(dat), ncol = nrep)
  # Take mode for each subject over nrep iterations
  class.dist$class <- as.numeric( apply(mat, MARGIN = 1,
            FUN=function(x){ names( sort( table(x), decreasing = T)[1])}))
  # Calculate proportion of time subject assigned to mode
  class.dist$prop <- apply(mat, MARGIN = 1,
            FUN=function(x){ max( table(x)) / length(x)})

  # Calculate Krippendorf's alpha
    # Measure of reliability of classification
  library(irr)
  print( kripp.alpha( t(mat), method = 'nominal'))

  # Return list
  return(list(class.dist, class.df))
}

ls <- classAssignment(dat, 50, 50)
```

```
##  Krippendorff's alpha
##
##  Subjects = 48
##    Raters = 50
##     alpha = 0.702
```

```
# Show example rows from output summary
head(ls[[1]])
```

```
##   class prop
## 1     8 0.62
## 2     2 0.96
## 3     3 0.78
## 4    17 1.00
## 5    17 0.76
## 6     6 0.66
```

```
# Write summary CSV file
write.csv( t( as.data.frame( matrix( unlist( lapply(
  ls[[2]], function(x) {x$class})), 48, 50),
  row.names = as.character( unique( merged$subject)))),
          file = 'confusionsBySub.csv', col.names = F, row.names = F)

# Print runtime for code chunk
difftime(Sys.time(),now)
```

```
## Time difference of 42.71146 mins
```

S3 Conclusions

The majority of subjects fell into the category with ideal distribution of means, but greater variance at lateral target angles. This pattern was not immediately apparent from the raw data and was not clear from LSI. Using unsupervised machine learning combined with bootstrap simulations of meaningful localization categories, it was possible to address a need for an objective classifier of localization performance on bilateral cochlear implant data. Moving forward, it will be possible to apply these techniques to relevant clinical populations.

Note that the approach described here can be applied to many different kinds of analysis problems in psychophysics, and generally to problems involving classification when theoretical groups are prescribed. In the above supplement, the terms ‘target angle’ and ‘response angle’ need only be changed to ‘independent variable’ and ‘dependent variable’, respectively. In order to extend our analysis approach to different kinds of psychophysical experiments in the future, we caution experimenters to define categories based upon a wide variety of literature, prefereably from different laboratories. We also encourage experimenters that extend our approach to adapt the code from the simulation study and ensure that the unsupervised machine learning algorithm is able to accurately classify data from your prescribed categories. In our manuscript, we outline some of the commonalities in localization data from listeners with bilateral cochlear implants, and the definition of categories was based upon a prior publication in our laboratory describing these categories.

Our analysis approach is limited in its utility by the accuracy of the categories we define in Figure **??**. We encourage experimenters to critique and refine these categories to maximize the utility of our analysis approach, and make the tool maximally beneficial to the field.
