# Supplementary material for: Sound source localization patterns and bilateral cochlear implants: Age at onset of deafness effects
Source: PLoS One. 2022 Feb 8;17(2):e0263516. doi: 10.1371/journal.pone.0263516 (PMC8824335; doi:10.1371/journal.pone.0263516)
Supplement: S2 Appendix — This appendix contains detailed mathematical descriptions of the algorithms used, as well as a simulation study detailing parameter choices, for the unsupervised machine learning analysis. (PDF) [file pone.0263516.s002.pdf]

Machine Learning Methods

Overview and Justification

The pattern of localization responses reveals important aspects of perception for listeners with BiCIs. When experimenters generalize across groups of patients with respect to their average error, they may be overlooking systematic errors made by each patient that are critical for everyday life. The choice to receive BiCIs in the first place may have been inspired by a desire to increase sound localization accuracy. Finally, if systematic errors are never identified, how can we hope to improve device design and patient outcomes?

Unsupervised machine learning was used to classify listeners' localization functions into different categories. There were many ways to conceptualize this problem, and attempts were made to compare against several other methods of analysis in the main manuscript (i.e., the RMS error across target angle and fitting data with a logistic curve). We chose to simulate many different cases of pre-defined localization functions and unsupervised machine learning because: (1) it simulates changes in the localization function that can occur from sampling, (2) it is extraordinarily flexible for the experimenter in that many different localization characteristics and shapes can be simulated, (3) classification can be completed with real or simulated data, (4) the output of the analysis is intuitive because meaningful differences in performance are defined before analysis is complete, (5) accuracy of classification with simulated data can be confirmed, (6) the analysis can be applied to datasets with few subjects and still remain accurate so long as sufficient number of repetitions are used, and (7) confusions between different classifications can be plotted and checked.

While we used an unsupervised machine learning algorithm to complete the analysis, it is similar in spirit to a supervised algorithm with voting based upon stochastic prototypes (e.g.,

[1]). One benefit of using an unsupervised machine learning algorithm during the analysis is that it can be applied to large, exemplary datasets where instead of simulating data from predefined categories, data from many different subjects (that do not have predefined categories) can be used. Unfortunately, the results in the present study suggest that the 48 patients that we tested do not provide sufficient examples of differences between listeners to complete such an analysis. It may be that more, exemplary listeners are needed to implement unsupervised machine learning based only upon experimental data in different kinds of experiments (e.g., those measuring psychometric functions), to which the unsupervised methods in the present study could be adapted.

In this appendix, we test properties of different implementations of the unsupervised machine learning approach to determine potential pitfalls and provide suggestions for future analyses.

Approach

The goal of our new analysis technique was to sort localization data into predefined, meaningfully different categories. The categories used in the present study are shown in Fig. 5 of the manuscript and the parameters for those categories are included in the R code that accompanies this paper. The choice of localization categories was guided primarily by Zheng and colleagues [2], who described prototypical error patterns that emerge in children with BiCIs. The present study contains a relatively large and rich dataset for listeners with BiCIs compared to existing literature. Thus, the choice of parameters used to capture categories was guided partly by data in the present study. The largest standard deviations that occur in Fig. 5 reflect some of the larger standard deviations that appear in the dataset. The choice of where the means of

localization across target angle begin to asymptote (near ±50 degrees) was guided by data in the present study and many previous studies [3–6].

Classification was completed using the different unsupervised machine learning algorithms by including the mean and standard deviation at each target angle as “features”. The output of the algorithm is a set of “clusters” which assign a listener and all simulated data to different categories. Accordingly, 50 subjects were simulated from each of the predefined categories illustrated in Fig. 5 with 15 repetitions per target speaker, as in the data from listeners. A total of $50 \times 20 = 1000$ simulated subjects and data from one listener were input to the unsupervised machine learning algorithm and classified into 20 different clusters. The mode of the category from the simulated data in the cluster containing the participant was taken for each classification. This process was repeated 50 times for each listener. The mode of the modes from 50 repetitions (including newly simulated data) was taken as the “true” category for that listener. As illustrated in Fig. 6, it was possible to show the frequency with which participants were classified into a particular cluster on each classification.

The simulation study below shows that with more data from few participants, greater accuracy can be achieved (when the category of the simulated cases is known). This analysis procedure is also utilizable for studies that include few data from few participants, but will result in higher rates of mis-classification due to sampling error. Performance from two different unsupervised machine learning algorithms was compared in the Simulation Study section.

Below is a brief description of each algorithm. Partitioning around medoids (PAM) was used for all analyses reported in the Results section of the manuscript based upon the results of the simulation study. Following these descriptions, results of the simulation study are shown.

Partitioning Around Medoids (PAM) Algorithm

The original description of the PAM algorithm is provided by Kaufman and Rousseeuw [7] and is outlined as implemented in the present experiment. An unsupervised learning algorithm attempts to minimize some dissimilarity criterion for a set of data where:

$$A = \begin{bmatrix} a_{11} & \cdots & a_{1f} \\ \vdots & \ddots & \vdots \\ a_{s1} & \cdots & a_{sf} \end{bmatrix} \quad \text{(A1)}$$

such that each element in the matrix A is a vector of f features with a total of s observations. Specifically, in this experiment there were $f = 38$ means or SDs at each target angle. During the analysis for each listener, 1000 simulated subjects and one listener were included in A, such that s = 1001. The PAM algorithm works by minimizing the distance between observations partitioned into clusters according to the following equation:

$$min \sum_{p=1}^{s} \sum_{q=1}^{s} d(p,q)\, z_{pq} \quad \text{(A2)}$$

where $d(p,q)$ is the dissimilarity between elements a_{pl} and a_{ql} for some $p,q = 1,\ldots,s$ and all $l = 1,\ldots,f$, and z_{pq} is a weight of 0 or 1 assigned to the pair of pth and qth observations. The following is true of z:

$$\sum_{p=1}^{s} z_{pq} = 1 \qquad for\ q = 1, \ldots, s \quad \text{(A3)}$$

subject to the constraint that z has exactly k rows containing non-zero elements, where k is the number of clusters specified by the experimenter. Thus, PAM is simply a specific case of a 0-1 linear programming problem. Each row in z corresponds to the inclusion of a particular observation into a set of “representative observations” used to compare against all other

observations. The ith column and jth row of z indicate the jth representative observation to which the ith observation is assigned (indicated by a value of 1). The representative observations are determined algorithmically.

The dissimilarity $d(p,q)$ is calculated as the pairwise Euclidian distance between all objects according to Eq. A4:

$$d(p,q) = \sqrt{\sum_{j=1}^{f} \left(a_{pj} - a_{qj}\right)^2} \qquad for\ p,q \in \{1, \ldots, s\} \quad \text{(A4)}$$

for the pth and qth observations of the jth features from the matrix A in Eq. A1. Alternative dissimilarity calculations can be specified by the experimenter if so desired.

The algorithm itself has two phases: initialization (i.e., BUILD) and changing of labels (i.e., SWAP). In the BUILD stage, representative observations are determined iteratively (and defined in z) beginning with the observation with the minimum dissimilarity to all other observations. For each following iteration, the dissimilarity between the ith observation and the most similar representative observation (D_i), as well as the ith observation and all other observations (D_j), is calculated. The difference between D_i and D_j will always be negative (since the first representative observation minimized dissimilarity). Once this calculation is completed for all s observations, the next representative observation is that which minimizes $D_i - D_j$. For iterations beyond the second, D_i may vary depending upon i since there is more than one representative observation in the set. This process is repeated until k representative observations are found, and these observations are used as the medoids for initial partitions.

In the SWAP stage, the dissimilarity between all representative and other observations, and for the case where the pth observation is included and its most similar representative nth

observation is excluded from the representative observations, is calculated as in the objective function in Eq. A5:

$$obj(p) = \sum_{q=1}^{s} d(p,q)\, z_{pq}^{*} - \sum_{q=1}^{s} d(p,q)\, z_{pq} \quad for\ p \in \{1, \ldots, s\} \quad \text{(A5)}$$

where values of z are chosen during the BUILD or previous SWAP stage and z^*_{pq} represents the case where the pth observation is included in the set of representative observations and its most similar, nth observation is excluded from that set. If the objective function is negative, then including the pth observation in the set of representative observations results in less dissimilarity or better performance, so a SWAP of the pth and nth observation is made. This results in the inclusion of the pth row of A as a representative observation and exclusion of the nth row of A. The SWAP process is completed for all observations. If no swaps are made for s iterations, then the algorithm stops.

K-Means Algorithm

Analysis was completed with another unsupervised algorithm called k-means. An early description of the k-means algorithm as implemented in this appendix is provided by Hartigan [8]. Here, we provide a brief overview of how the algorithm works using the same matrix A from Eq. A1. The k-means algorithm minimizes the distance between points within a given number of k clusters specified by the user and their centroids according to Eq. A6:

$$\min \sum_{p=1}^{s} d(p) \quad \text{(A6)}$$

where $d(p)$ is the dissimilarity between the pth row of A and the mean of each cluster in A (see Eq. A7).

One aspect that distinguishes k-means from PAM is that dissimilarity is the Euclidian distance or root-sum-squared difference between all observations *in a cluster* and the *mean of that cluster*. In contrast, dissimilarity in PAM is calculated by taking the difference between representative observations and *all other observations*. Thus, the dissimilarity in k-means is defined in Eq. A7:

$$d_u(p) = \sqrt{\sum_{j=1}^{f} (a_{pj} - \underline{a}_{u_j})^2} \qquad for\ u \in \{1, \dots, k\} \quad \text{(A7)}$$

where $d_u(p)$ is the error for the pth observation partitioned into cluster u over each jth feature, and $\bar{a}_{uj}$ represents the mean of the jth feature in cluster u.

The k-means algorithm works by randomly assigning all s observations to a particular cluster k and computing the dissimilarity (Eq. A6). For the pth observation previously assigned into cluster u, an objective function is calculated from Eq. A8:

$$obj(p) = \left(\frac{n_t\, d_t(p)^2}{n_t + 1} - \frac{n_u\, d_u(p)^2}{n_u - 1} \right) \qquad for\ u \in \{1, \dots, k\}\ and\ t = 1, \dots, k \neq u \quad \text{(A8)}$$

where n_t and n_u are the numbers of observations in clusters t and u, respectively, and d_t and d_u represent the dissimilarity for the pth observation partitioned into clusters t and u, respectively. If obj(p) for any t is negative, then the pth observation should be moved from cluster u to cluster t where the objective function reaches its minimum, and $\underline{a}_{u_j}$, $\underline{a}_{t_j}$, $d_t(p)$, and $d_u(p)$ should be recalculated iteratively for p=1,...,s. After s iterations for each observation, if the error did not change (i.e., no observations were partitioned into new clusters), then the algorithm stops.

Simulation Study

It is plausible that PAM is more robust than k-means because it relies on pairwise dissimilarity between all and representative observations, compared to k-means, which relies on

only differences within a single cluster and the cluster mean. This hypothesis was tested in a simulation study to guide the choice of which algorithm to use for the new analysis approach.

There are two types of validation tools that exist for unsupervised machine learning algorithms: internal and external. Internal validation refers to the process of examining clusters to determine similarity within clusters and can be completed without knowing the "true" category to which a particular observation belongs. External validation refers to the process of examining clusters for accuracy based upon the "true" categories. It is possible to perform external validation in simulation studies where performance of the algorithm is assessed based upon how accurately a simulated data are sorted into the categories provided, and there is a correct category to which each simulated individual belongs. In this simulation study, external evaluation was used to compare the performance of these two unsupervised machine learning algorithms.

In so doing, it was possible and important to confirm that the procedure described in the Approach section above yielded accurate performance and determine what parameters in the data that affect performance. Therefore, the same technique was used as in the Approach, but no data from actual listeners were used. Instead, only simulated data were used to evaluate algorithm performance. This process was completed 2000 times with a newly simulated dataset. It was then possible to determine how often the procedure resulted in an assignment of simulated categories to the correct assignment (from Fig. 5). Three metrics were used as indices of algorithm performance and are included in the R code that accompanies the present paper: purity, normalized mutual information, and Rand index.

Cluster purity is a statistic that represents the number of clusters containing the same category and is defined in Eq. A9:

$$\text{Purity} = \frac{1}{s}\sum_{p=1}^{s}\left|u_p \cap t_p\right| \qquad for\ u\ and\ t \in \{1, \ldots, k\} \quad \text{(A9)}$$

where s represents the total number of observations or simulated subjects, for the true and estimated categories u and t for the pth observation or row in A, and $|U|$ indicates the number of items in a set U. That is, when the true and estimated categories match, the expression in the summation takes on a value of 1, and when they do not match, it takes on a value of 0, for a total of s observations. Thus, purity indicates the consistency of clustering averaged across all clusters u_p.

The values that u and t take on over all observations can be summarized by the cluster number 1,…,k to form k sets of $\{c_{(1)},\ldots,c_{(k)}\} \in C$ and $\{b_{(1)},\ldots,b_{(k)}\} \in B$, representing the observations or rows in A to which each observation belongs or was algorithmically assigned, respectively.

Mutual information was first discussed by Shannon [9], is calculated by comparing the overlap between the true and algorithmically estimated classes, and is defined in Eq. A10:

$$\text{Mutual Information} = \sum_{i=1}^{k}\sum_{j=1}^{k}\frac{|c_{(i)} \cap b_{(j)}|}{s}\log\frac{s\,|c_{(i)} \cap b_{(j)}|}{|c_{(i)}|\,|b_{(j)}|} \quad \text{(A10)}$$

where $c_{(i)}$ and $b_{(j)}$ are the sets of observations or rows in A for the ith true and jth algorithmically estimated categories, respectively. Mutual information is bounded between (0, ∞), the upper-limit of which is determined by the entropy of partitions $C = \{c_{(1)},\ldots,c_{(k)}\}$ and $B = \{b_{(1)},\ldots,b_{(k)}\}$. To convert to normalized mutual information, which is bounded between 0 and 1, mutual information is divided by the maximum of the entropy of C and B, and the expected value is subtracted from the numerator and denominator.

Finally, the Rand index is given by Rand [10], is calculated by taking the probability of choosing a correct clustering (including and excluding observations accurately), and is defined in Eq. A11:

$$\text{Rand Index} = \frac{1}{\binom{s}{2}} \sum_{p<q}^{s} \gamma_{pq} \quad \text{(A11)}$$

$$\text{where } \gamma_{pq} = \begin{cases} 1 \text{ if there exist } i \text{ and } j \text{ such that both the } p\text{th and } q\text{th row of } A \text{ are in both } c_{(i)} \textit{ and } b_{(j)} \\ 1 \text{ if there exist } i \text{ and } j \text{ such that the } p\text{th row of } A \text{ is in both } c_{(i)} \text{ and } b_{(j)} \text{ while the } q\text{th row of } A \text{ is in neither } c_{(i)} \text{ nor } b_{(j)} \\ 0 \text{ otherwise} \end{cases} \quad \text{for } i, j = 1, \dots, k$$

and $c_{(i)}$ and $b_{(j)}$ are defined as in Eq. A10, and s is the number of simulated subjects. Thus, the Rand index is a ratio of the sum of true positives and negatives over the total number of possible combinations.

The number of repetitions per target speaker was also varied to determine how this affected clustering purity, normalized mutual information, and rand index. Each of these measures is bounded between 0 and 1 and gives a measure of classification accuracy. A value of 1 indicates perfect performance of the unsupervised machine learning algorithm. These data are summarized in Fig. A1 for each measure. Fig. A1 shows that for all but the fewest repetitions per target speaker, PAM performed as well as or better than k-means on each external evaluation measure, especially when more simulated subjects were included in the analysis. PAM and k-means performed better with more repetitions per target speaker were included, and PAM surpassed k-means around 15-20 repetitions per speaker for 50-100 simulated subjects.

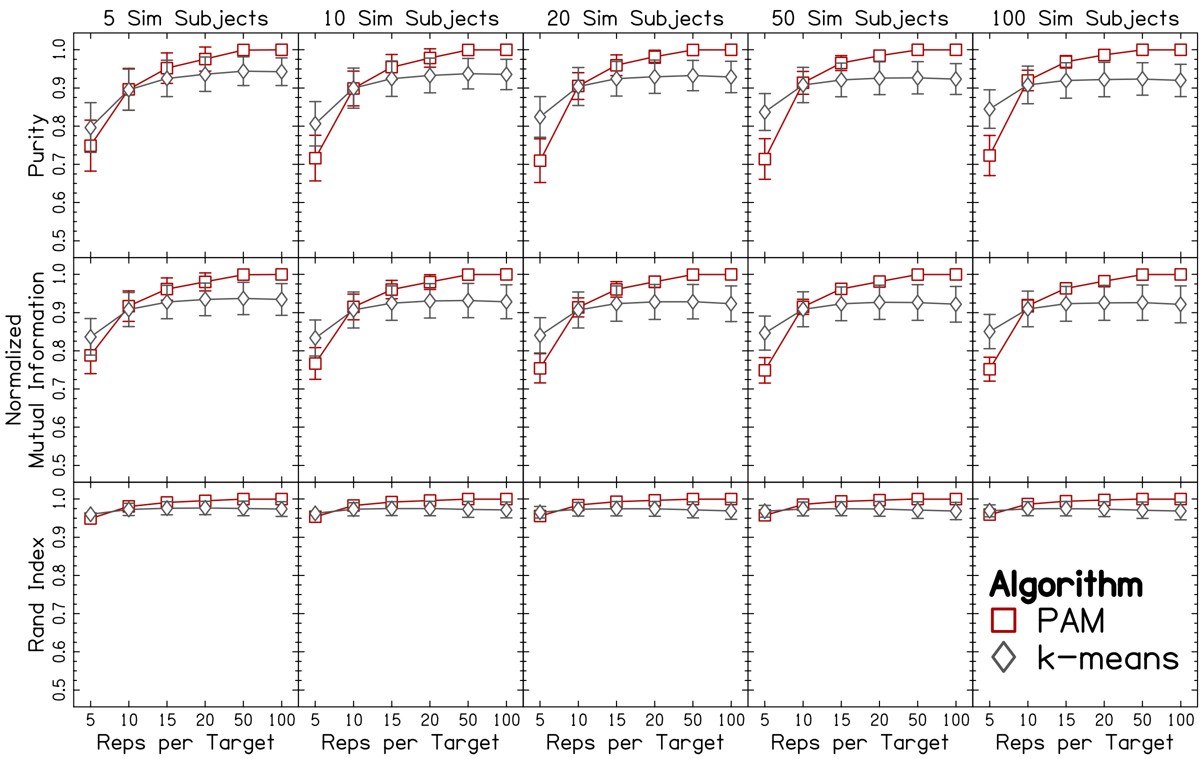

Fig. A1. External evaluation of PAM and k-means performance for simulated data. Data were simulated based upon the categories in Fig. 5. Each column represents a different number of simulated subjects per category in Fig. 5. Each row represents a different external evaluation criterion. The x-axis shows the number of repetitions simulated per target angle (i.e., for the 19 target angles that appear in each panel of Fig. 5). The y-axis shows the magnitude of each criterion of performance. Error bars represent 95% confidence intervals about the mean. Confidence intervals were calculated by taking the mean of the criterion across simulations and adding the z-score for 95% probability density (1.960) times the standard deviation from the 2000 samples. The standard deviation was used instead of standard error because the confidence interval is meant to reflect the distribution from the simulations. All criteria were bounded between 0 and 1, where 1 indicates perfect performance. Data from PAM are shown in red and data from k-means are shown in grey.

Clusters in Dataset

Another possible approach to the problem would have been to include all data from participants to determine the number of different sound source localization functions that were apparent in the present data. To address this question, instead of including simulated data, data from all participants were included in PAM and internal validation criteria were used to determine the number of potential clusters in the dataset. PAM was used instead of k-means based upon the simulation study results.

The procedure described by Hennig and Lin [11] was used to determine the ideal number of clusters. Recall that the number of clusters must be specified with the input of PAM, so this procedure provides one way to estimate the ideal number of clusters in a particular dataset. Briefly, the procedure relies on bootstrap simulations of data with no clustering (i.e., a null model) and a comparison against data input to PAM. Experimental data were placed in a matrix, where each row corresponded to a different subject, and each row corresponded to the mean or SD of response angles for that subject (i.e., "features" for a clustering algorithm) as in Eq. A1. Bootstrap simulations were completed by simulating from a multivariate standard normal distribution, estimating the correlation matrix from the mean and SD at each target angle across listeners with BiCIs, and applying the inverse normal transformation using the means from experimental data. The resulting bootstrap simulations thus had a similar pattern of means and covariance but with no clustering. The results of this analysis are shown in Fig. A2. The results indicate that the average silhouette width (an internal validation criterion used to determine consistency within clusters where higher values indicate better performance) falls within the 99% confidence interval surrounding the null model with three clusters included in PAM. The upper bounds of confidence intervals were calculated by taking the mean of average silhouette widths

across simulations and adding the z-score for 99% probability density (2.575) times the standard deviation from the 500 samples. The standard deviation was used instead of standard error because the confidence interval is meant to reflect the distribution from the simulations, not the distribution of means from some theoretical probability density function associated with the null model.

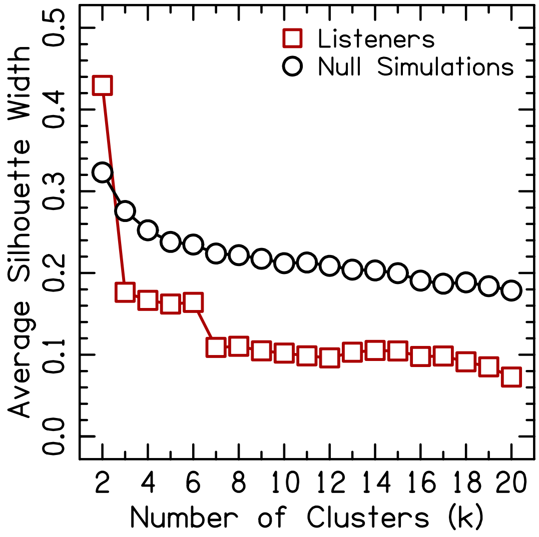

Fig. A2. Estimation of number of clusters in dataset. This type of analysis is based on that proposed by Hennig and Lin [11]. Five hundred simulations of a null model containing no clustering were used to determine the confidence intervals around average silhouette width. The x-axis shows the number of clusters estimated by PAM. The y-axis shows the average silhouette width, where higher values indicate greater similarity within, and greater differences between, clusters. Average silhouette width from the data collected in the experiment are shown in red and data from the null model bootstrap are shown in black.

Results from Fig. A2 suggest that there were only two clusters that emerge naturally in the dataset from the present study. This result may at first glance suggest to the reader that the

analysis procedure employed in the present study was inappropriate. However, large variability across listeners with BiCIs is typical in the literature. Though 48 listeners is considerably large for BiCI studies, it may not provide a sufficient number of examples of the kinds of performance differences that can be observed across listeners. It may also be that the listeners participating in the present study were not sufficiently representative of differences between listeners with BiCIs in the literature. The use of data from simulated categories avoids this problem and makes it possible to complete this type of analysis when few listeners with BiCIs participate in a study, a commonality in the literature. Further, using simulated data will allow for fairer comparisons between future studies that might adopt this procedure.

References

1. Li O, Liu H, Chen C, Rudin C. Deep learning for case-based reasoning through prototypes: A neural network that explains its predictions. arXiv Prepr. 2017;arXiv:1710.04806.
2. Zheng Y, Godar SP, Litovsky RY. Development of sound localization strategies in children with bilateral cochlear implants. PLoS One. 2015;10(8):e0135790.
3. Grantham DW, Ashmead DH, Ricketts TA, Labadie RF, Haynes DS. Horizontal-plane localization of noise and speech signals by postlingually deafened adults fitted with bilateral cochlear implants. Ear Hear. 2007;28(4):524–41.
4. Nopp P, Schleich P, D'Haese P. Sound localization in bilateral users of MED-EL COMBI 40/40+ cochlear implants. Ear Hear. 2004;25(3):205–14.
5. van Hoesel RJM, Tyler RS. Speech perception, localization, and lateralization with bilateral cochlear implants. J Acoust Soc Am. 2003;113(3):1617–30.
6. Jones H, Kan A, Litovsky RY. Comparing sound localization deficits in bilateral cochlear-implant users and vocoder simulations with normal-hearing listeners. Trend Hear. 2014;18:1–16.
7. Kaufman L, Rousseeuw PJ. Clustering by means of medoids. In: Dodge Y, editor. Statistical Data Analysis Based on the L1 Norm and Related Methods. Amsterdam, The Netherlands; 1987. p. 405–16.
8. Hartigan JA. The K-means algorithm. In: Clustering Algorithms. New York, NY: John Wiley & Sons, Ltd.; 1975. p. 84–107.
9. Shannon CE. A mathematical theory of communication. Bell Labs Tech J. 1948;27(3):379–423.

10. Rand WM. Objective criteria for the evaluation of clustering methods. J Am Stat Assoc. 1971;66(336):846–50.

11. Hennig C, Lin CJ. Flexible parametric bootstrap for testing homogeneity against clustering and assessing the number of clusters. Stat Comput. 2015;25:821–33.
